# Myasthenia thymus reprograms class-switched B cells into BAFF-dependent survivors

**DOI:** 10.1126/sciadv.aec3842

**Published:** 2026-08-12

**Authors:** Yuanqing Yan, Diego Avella Patino, Yimeng Zhao, Xin Wu, G. R. Scott Budinger, Ankit Bharat

**Affiliations:** ^1^ Department of Surgery, Division of Thoracic Surgery, Feinberg School of Medicine, Northwestern University, Chicago, IL 60611, USA.; ^2^ DeWitt Daughtry Family Department of Surgery, Miller School of Medicine, University of Miami, Coral Gables, FL 33124, USA.; ^3^ Department of Medicine, Division of Pulmonary and Critical Care Medicine, Feinberg School of Medicine, Northwestern University, Chicago, IL 60611, USA.

## Abstract

Myasthenia gravis (MG) presents a clinical challenge where autoantibody titers poorly predict disease severity, and thymectomy provides inconsistent benefits. We hypothesized that thymic B cells acquire survival mechanisms that bypass canonical tolerance checkpoints, enabling persistence independent of antigen-specific selection. Using single-cell RNA sequencing, V(D)J repertoire analysis, and spatial transcriptomics to generate the largest MG atlas to date (237,661 cells), we identified a tolerance checkpoint swap in MG pathogenesis. Pathological class-switched B cells within thymic germinal centers exhibited reduced antigen presentation (*CD74* and *CD1C*) and diminished *CD40* costimulation while up-regulating *TNFRSF17* to engage BAFF-driven survival. This shift allows polyclonal autoreactive B cells to evade stringent selection and seed peripheral sites. T follicular helper cells supported this reprogramming via increased *TNFSF13B* and *CHGB* expression, with the latter facilitating dopamine-mediated synaptic acceleration. These findings offer insight into clinical paradoxes in MG and point to the BAFF-BCMA axis as a potential therapeutic target and biomarkers for disease activity.

## INTRODUCTION

Myasthenia gravis (MG) represents a clinical paradox in which pathogenic autoantibodies against the neuromuscular junction drive muscle weakness and fatigability, yet antibody titers correlate poorly with disease severity and treatment response (*1*–*4*). Even more puzzling, thymectomy provides inconsistent clinical benefit despite being a mainstay of treatment. This surgical removal of the thymus where autoreactive B cells are known to originate should eliminate the source of pathogenic antibodies, yet many patients continued to produce these autoantibodies. These clinical observations challenge our understanding of how autoimmune conditions such as MG are sustained and underscore the inefficacy of current therapeutic approaches, which focus primarily on antibody reduction.

Thymus plays a central yet incompletely understood role in MG pathogenesis. Beyond its classical function in T cell education, the MG thymus can reveal notable pathological changes. Thymic hyperplasia or thymoma occurs in up to 85% of patients (*5*). Ectopic germinal centers (GCs) form within the medulla, and specialized thymic epithelial cells aberrantly express neuromuscular antigens (*2*, *3*). These features point to the thymus not merely as a site of initial tolerance breakdown but as an active driver maintaining autoimmunity through mechanisms that extend beyond antigen presentation. The persistence of disease despite thymectomy and the disconnect between antibody levels and clinical severity suggest that thymic B cells may acquire survival advantages that allow them to persist independent of conventional selection pressures.

Recent work has identified neuromuscular thymic epithelial cells (nmTECs) that express acetylcholine receptor (AChR) components and highlighted innate immune signatures in MG thymus (*2*, *3*, *5*). However, these findings alone cannot explain why some B cells escape normal tolerance checkpoints while others do not. They also cannot explain how autoreactive B cells maintain themselves despite therapeutic interventions. Understanding the molecular programs that enable B cell survival within thymic microenvironments could reveal why conventional treatments fail and identify new therapeutic vulnerabilities.

Here, we used a human tissue–anchored integrated multiomic approach to uncovering a previously unrecognized mechanism of tolerance breakdown. We combined single-cell RNA sequencing (scRNA-seq), V(D)J repertoire analysis, and spatial transcriptomics from the largest MG thymic cohort assembled to date. We suggest that MG pathogenesis involves a fundamental reprogramming of B cell survival. Specifically, we identified a shift from classical T cell–dependent activation requiring CD40 costimulation to an innate-like program driven by BAFF (*TNFSF13B*)–BCMA (*TNFRSF17*) signaling. This tolerance checkpoint swap occurs within spatially defined GCs and enables diverse autoreactive B cells to survive without stringent antigen–specific selection. Our findings offer a potential explanation for longstanding clinical dilemmas in MG and identify promising therapeutic targets for restoring immune tolerance.

## RESULTS

### Patient characteristics

From a total collection of 23 samples (16 patients; table S1), 18 samples were used for this study: thymus without MG (*n* = 4), thymus with MG (*n* = 8), thymoma without MG (*n* = 3), and thymoma with MG (*n* = 3). The cohort had a mean age of 48.1 years (range: 21 to 78) and a male-to-female ratio of 5:11. Some patients contributed more than one sample. For example, patient PT4 provided samples of both thymus without MG and thymoma without MG. To enhance our analytical power, we also included scRNA-seq data from two external studies: thymoma with MG samples from Yasumizu *et al.* (*3*) (includes cells from both peripheral blood and thymoma tissues) and thymic epithelial tumor samples from Xin *et al.* (*6*). In total, this study included 346,987 cells, of which 237,661 were from our own cohort.

### Single-cell data integration and *AChR* gene expression in thymic epithelial cells

We initiated our analysis by conducting data integration using scvi-tools (v1.1.6.post2) and identified the cell clusters using the Leiden clustering (fig. S1A) (*7*). To evaluate the performance of the integration, we used the scIB package (v1.1.7) to assess both batch effect removal and biological conservation (*8*). The scVI integration achieved a high graph connectivity score of 0.87, which is comparable to (and slightly higher than) the 0.86 achieved by Harmony, confirming successful batch effect removal while preserving biological heterogeneity. Furthermore, a normalized mutual information (NMI) value of 0.70 demonstrated that the integration effectively preserved the underlying biological structure of the data. We also examined cell distribution bias across datasets within clusters, variations in gene and Unique Molecular Identifier (UMI) counts among clusters, and the segregation of cells into distinct populations (fig. S1, B and C). These analyses confirmed that the integration process did not introduce notable biases associated with dataset differences.

On the basis of the cell compartment marker genes, we successfully identified the major cellular compartment, including epithelial (*EPCAM*^+^), endothelial (*PECAM1*^+^), immune (*PTPRC*^+^), and stromal cells (fig. S1C). Focusing on structural cells, we leveraged the cell type–specific gene markers to identify 11 epithelial, 6 endothelial, and 3 stromal cell types (fig. S2A). Notably, among epithelial cells, we were able to detect rare populations, such as thymic myoid, ciliated, and ionocyte cells from our analysis.

Using the annotated epithelial cells, we investigated the presence of nmTECs, a pathological cell type critical for the ectopic expression of neuromuscular molecules in thymoma with MG, as described by Yasumizu *et al.* (*3*). We matched cell barcodes annotated for this cell type and found that nmTECs did not form a unique cluster but were instead embedded within the KRT15^+^ mTEC population (fig. S2B). In addition, we analyzed the expression of genes encoding AChR and found no significant differences between MG and non-MG (NoMG) samples (figs. S2C and S3). These observations suggest that mechanisms beyond self-antigens may contribute substantially to the pathogenesis.

### Altered B cell immune response in patients with MG revealed by pseudobulk RNA-seq analysis

**Fig. 1.**
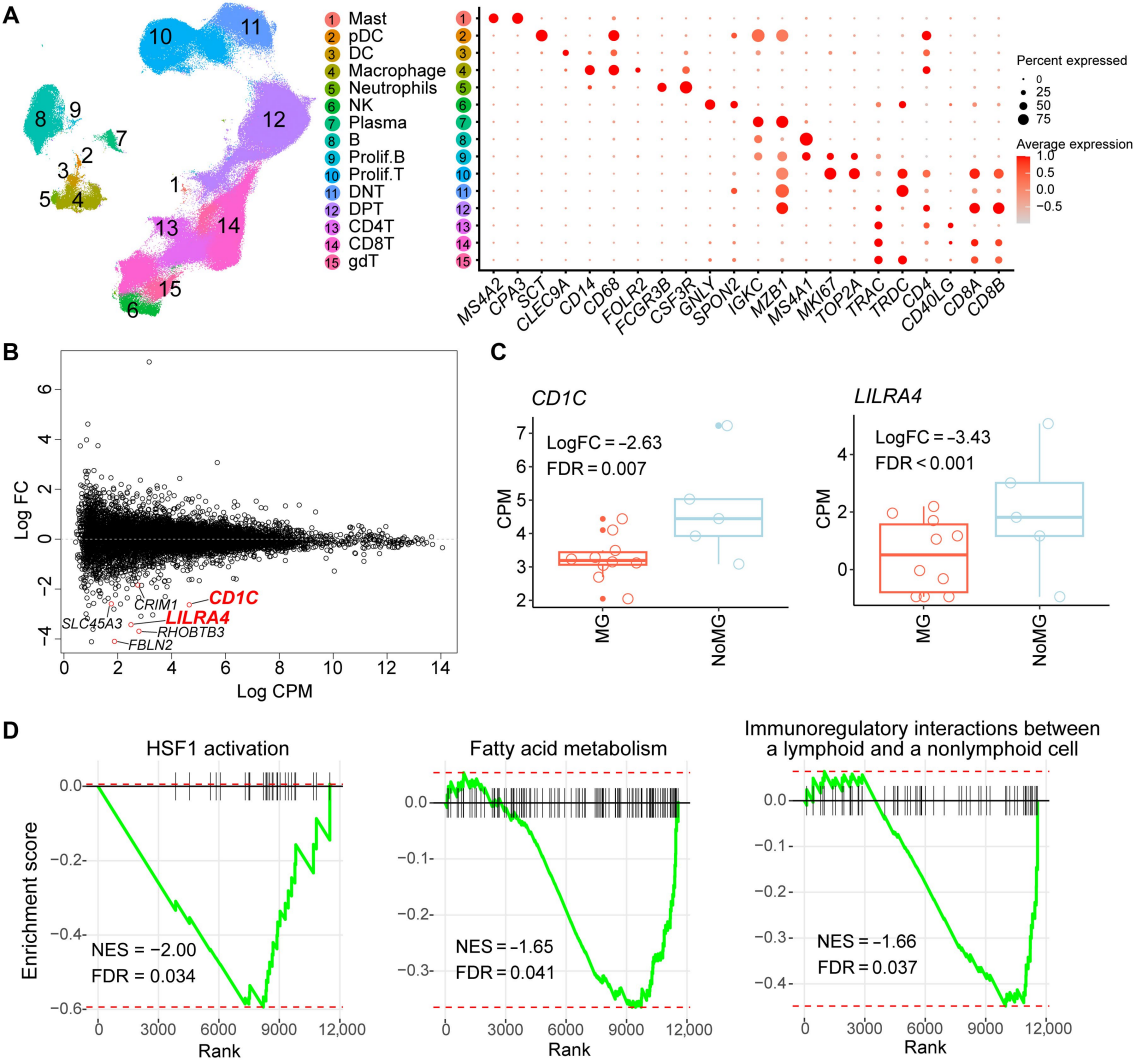
Altered molecular profile in B cells of patients with MG revealed by pseudobulk RNA-seq. (**A**) UMAP (left) and dot plot (right) illustrating the identification of immune compartment cell types. Within the B cell lineage, distinct clusters of B, plasma, and Prolif.B were identified. (**B**) MA plot (minus-average plot) delineating differentially expressed genes between MG and NoMG groups in the pooled B cell compartment (comprising B and Prolif.B cells). Pseudobulk RNA-seq profiles were generated by aggregating raw single-cell counts from both B and Prolif.B clusters per subject. Differential expression was modeled using the edgeR package (v4.2.2), adjusting for disease status and sequencing chemistry. *P* values were adjusted using the Benjamini-Hochberg (BH) procedure, with an FDR < 0.05 and absolute log_2_ fold change (FC) > 1 considered statistically significant. Each dot represents a gene; the *x* axis indicates log_10_ counts per million (CPM) and the *y* axis indicates log_2_FC. (**C**) Box plots showing the differential expression of *CD1C* and *LILRB4* genes between MG and NoMG samples. Each dot represents an individual subject (*N* = 10 MG; *N* = 5 NoMG), and whiskers represent 1.5× the interquartile range. (**D**) GSEA rank plot demonstrating enrichment of three significant pathways in the NoMG samples. Analysis was performed using the fgsea package (v1.30.0) with the MSigDB (v7.5.1) Reactome reference database. Negative normalized enrichment scores (NES) indicate pathway enrichment in the NoMG group. *P* values were adjusted by the BH method with an FDR < 0.05 considered statistically significant. pDC, plasmacytoid dendritic cells; DC, dendritic cells; NK, natural killer cells; Prolif.T, proliferating T cells; DNT, double-negative T cells (CD4^−^CD8^−^); DPT, double-positive T cells (CD4^+^CD8^+^); gdT, gamma-delta T cells.

Since MG is a B cell–mediated autoimmune disease, we started our analysis from the transcriptomic profile changes in B cells. Using established immune cell gene markers, we identified the B lineage within the immune compartment: B cells, plasma cells, and proliferating B cells (Prolif.B) (Fig. 1A). To compare MG and NoMG groups, we applied a pseudobulk analysis with offsets, which offers a scalable and computationally efficient alternative to generalized linear mixed models for controlling the false discovery rate (FDR) while maintaining comparable statistical performance (*9*, *10*). Our pseudobulk RNA-seq analysis identified six significantly down-regulated genes in patients with MG (Fig. 1B). Notably, *CD1C*, a gene responsible for antigen presentation and interaction with other immune cells (e.g., T cells), was significantly down-regulated (Fig. 1C). This suggests that patients with MG exhibited reduced immune regulation due to dampened B cell–mediated antigen presentation. In addition, we found that *LILRA4*, an inhibitory receptor that dampens immune responses via binding with ligands on other cells (*11*), was also significantly down-regulated.

Subsequent gene set enrichment analysis (GSEA) revealed that multiple biological pathways were significantly dysregulated in patients with MG (Fig. 1D). Specifically, the heat shock factor 1 activation pathway was down-regulated in the MG group. This pathway is important for the development of regulatory T cell (T_reg_ cell), which help to control inflammation (*12*). We also found a reduction in the fatty acid metabolism pathway. Furthermore, the observed down-regulation of immunoregulatory interactions between lymphoid and nonlymphoid cells in the MG group provides additional evidence of disrupted B cell–mediated cellular interactions.

### Identification of pathological class-switching B cells in patients with MG

**Fig. 2.**
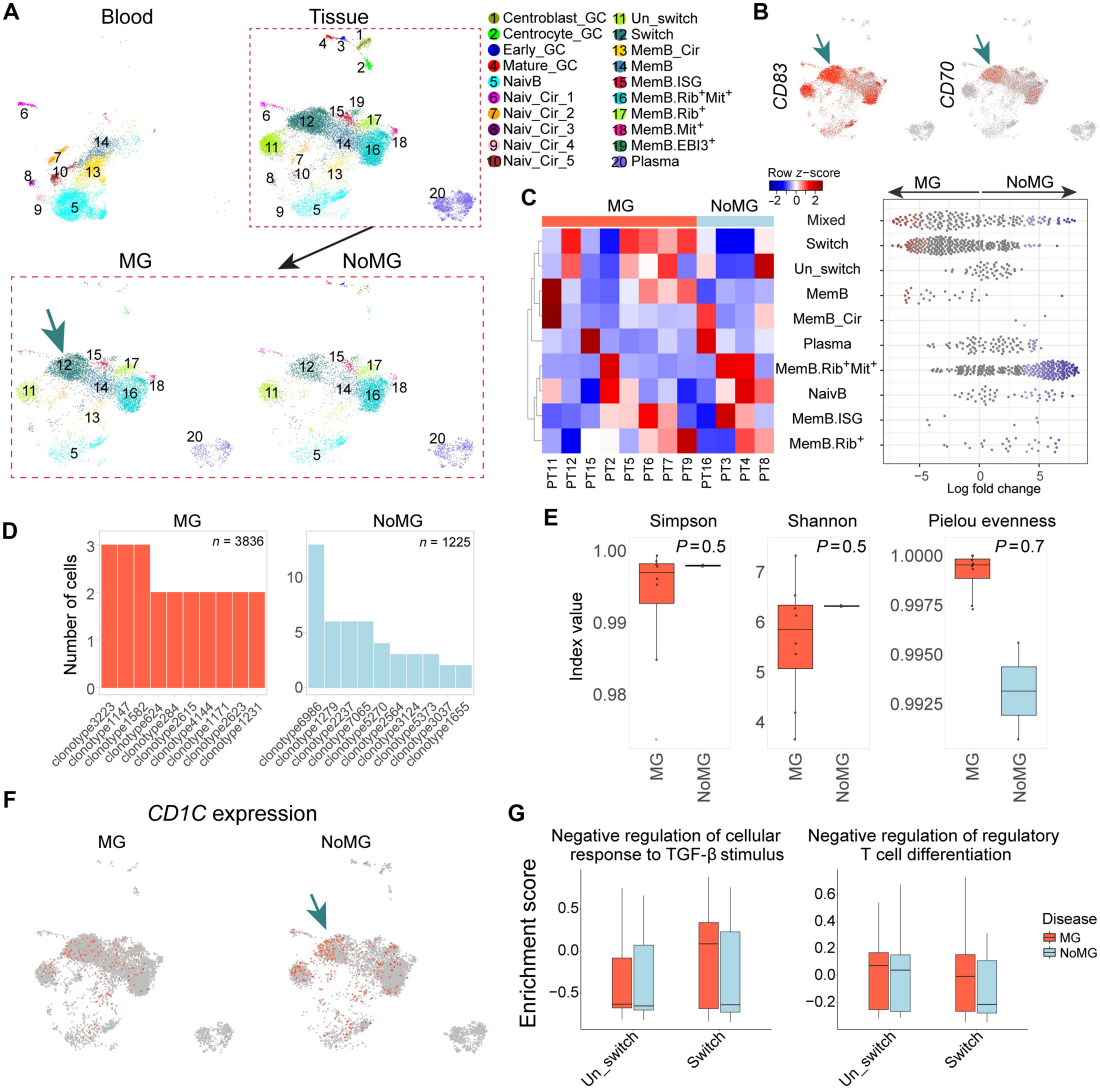
Identification of pathological class-switched B cells in patients with MG. (**A**) UMAP visualization delineating the enrichment of Switch B cells in patients with MG. Twenty distinct subtypes were defined; UMAPs are split by organ and disease status to illustrate subtype distribution across conditions. (**B**) Feature plots showing the overexpression of *CD83* and *CD70* in the Switch B cells. (**C**) Heatmap and beeswarm plot demonstrating the increased abundance of Switch B cells in MG. Proportions were calculated as a percentage of total cells per patient and row *z*-score normalized for heatmap visualization. The beeswarm plot was generated using miloR (v2.0.0), with cell types assigned to neighborhoods based on majority frequency (>0.7); the “mixed” population represents neighborhoods with no single dominant cell type. (**D**) Bar plot showing the abundance of the top clonotypes within the B cell lineage in MG (*n* = 3836 cells) versus NoMG (*n* = 1225 cells). (**E**) Boxplots comparing B cell receptor repertoire diversity between patients with MG (*n* = 8) and NoMG patients (*n* = 2) using three metrics of Simpson index, Shannon entropy, and Pielou’s evenness. Diversity indices were estimated using the djvdj package (v0.1.0), and statistical significance was evaluated via a two-sided Student’s *t* test. Individual data points represent distinct patient samples; whiskers represent 1.5× the interquartile range, and the horizontal line represents the median. (**F**) Feature plot illustrating the down-regulation of *CD1C* in the Switch B cells, corroborating pseudobulk RNA-seq findings. (**G**) Box plot illustrating the up-regulation of two biological process pathways in Switch B cells from MG samples. Enrichment scores were calculated via Seurat’s AddModuleScore; significance was determined using Wilcoxon rank sum test (FDR < 0.001). NaivB, naïve B; Naiv_cir_1, naïve circulating B; MemB, memory B; MemB_Cir, circulating memory B; MemB.Rib^+^Mit^+^, ribo-mitochondrial high memory B; MemB.EBI3^+^, EBI3-expressing memory B; MemB.ISG, interferon-stimulated gene-expressing memory B; Un_switch, immunoglobulin class-unswitched B; Switch, immunoglobulin class-switched B.

Given the heterogeneity of the B cell lineage, we further examined B cell subtypes using subclustering analysis. This revealed three major subtypes—germinal center B cells, nongerminal center B cells, and antibody-secreting cells—defined by the expression of *CCR7*, *CD38*, and *PRDM1* (fig. S4A). We then performed fine cell type annotation using cell type–specific markers, identifying a total of 20 distinct B cell subtypes with different enrichment between peripheral blood and tissue, consistent with the findings of Yasumizu *et al. (*3**) (Fig. 2A and fig. S4, A to D). Among these, two subtypes were particularly interesting: the immunoglobulin-class unswitched (labeled as Un_switch) and switched (labeled as Switch) subtypes. The Un_switch resembled a naïve state, with overexpression of the naïve marker *TCL1A*, along with high levels of *IGHD* and *IGHM* (fig. S4, B and D). In contrast, the Switch subtype exhibited high levels of *IGHA1* and overexpression of the memory marker *CD27*. Moreover, both the Un_switch and Switch subtypes are activated B cells, characterized by the overexpression of activation markers *CD83* and *CD69* (Fig. 2B and fig. S4B). A key molecule that distinguishes these two subtypes is the overexpression of *CD70* in the Switch subtype (Fig. 2B), which fosters B-T cell interactions and drives B cell maturation and differentiation, including plasma cell generation. Notably, both subtypes were detected exclusively in tissue samples but not in peripheral blood (Fig. 2A).

By splitting our tissue data into MG and NoMG groups, our Uniform Manifold Approximation and Projection (UMAP) revealed a marked expansion of the Switch subtype in patients with MG relative to the Un_switch subtype (Fig. 2A). Cell type proportional analysis, which calculated the proportion of each B cell subtype per sample, confirmed the increased abundance of the Switch subtype in patients with MG, despite some patient-to-patient variation (Fig. 2C and fig. S4, E and F). To statistically validate this enrichment, we used miloR (v2.0.0) for differential neighborhood abundance testing (*13*), which confirmed a significant enrichment of the Switch subtype within the MG group (Fig. 2C). Collectively, these results suggest that the Switch subtype is a key cellular component of the MG microenvironment and may contribute to disease pathogenesis.

Furthermore, our integrated dataset enables tracking of different B cell subtypes across both tissue and peripheral blood. We observed the coexistence of memory B cell subsets, marked by high *CD27* expression, in both compartments (figs. S4B and S5). In the Northwestern University (NU) cohort, RNA velocity analysis indicated that Switch B cells likely serve as precursors to these memory B cells (fig. S5). This suggests that Switch B cells can mature into memory B cells within GCs and that some memory B cells may reside in tissue while also circulating in the blood.

### B cell clonal diversity and dysregulated molecular profiles in MG

To further characterize B cell dynamics, we examined clonal expansion patterns using V(D)J sequencing. Clonotype analysis revealed no dominant B cell clone enriched in MG samples, as indicated by the small number of cells within each top clonotype (Fig. 2D). Repertoire diversity analysis showed comparably high clonotype diversity in the B cell lineage of both patients with MG and NoMG patients, without evidence of clonal expansion (Fig. 2E). This suggests that MG pathology is not driven by a single dominant clone but rather by dysregulated interactions within a diverse B cell pool.

We next investigated molecular differences between MG and NoMG samples. We first confirmed the down-regulation of *CD1C*, an antigen presentation gene identified in our pseudobulk RNA-seq analysis, with notably lower expression in the Switch subtype of patients with MG (Fig. 2F and fig. S4G). Using Gene Ontology:Biological Process as the reference, we performed pathway enrichment analysis and identified pathways significantly dysregulated between MG and NoMG within the Switch subtype. Notably, the negative regulation of cellular response to transforming growth factor–β (TGF-β) stimulus pathway was significantly activated in MG (Fig. 2G), suggesting a loss of tight regulation over the TGF-β pathway, which is critical for suppressing autoimmunity. The negative regulation of T_reg_ cell differentiation pathway was also activated in patients with MG. Together, these findings indicate that dysregulated T-B cell interactions, particularly involving the Switch subtype, may contribute to the breakdown of immune tolerance and promote autoantibody production.

### Altered expression profile of T follicular helper cells in patients with MG

**Fig. 3.**
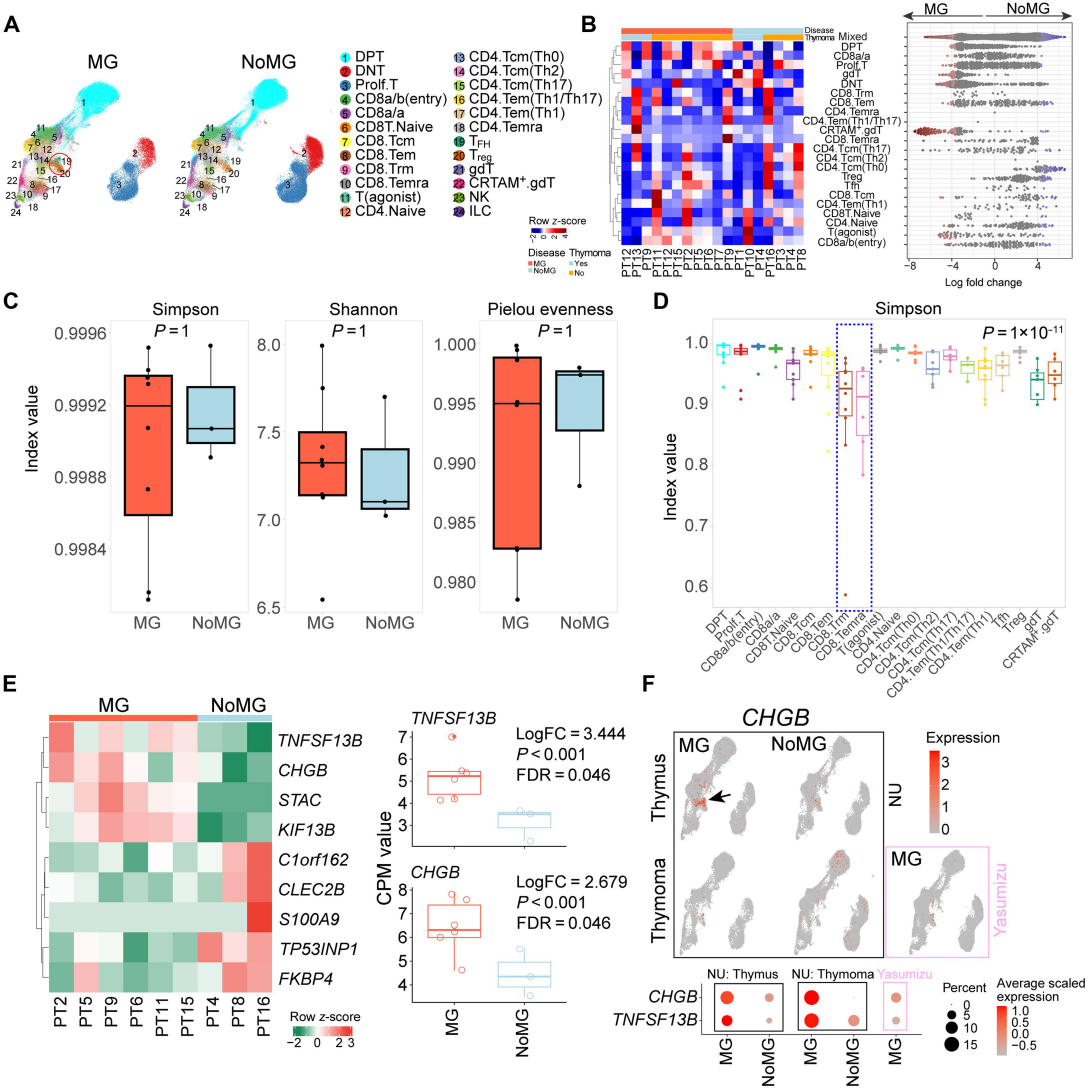
T cell clonal diversity and up-regulation of *TNFSF13B* and *CHGB* in MG T_FH_ cells. (**A**) UMAP visualization of T cell subtypes split by disease status. (**B**) Heatmap and beeswarm plot demonstrating the comparable abundance of multiple T subtypes between MG and NoMG samples. Proportions were calculated as a percentage of total cells per patient and row *z*-score normalized for heatmap visualization. The beeswarm plot was generated using miloR, with neighborhoods assigned on the basis of majority frequency (>0.7); the mixed population represents neighborhoods with no single dominant cell type. (**C**) T cell receptor (TCR) repertoire diversity comparison (Simpson index, Shannon entropy, and Pielou’s evenness) between MG (*n* = 8) and NoMG (*n* = 3) using the djvdj package. Significance was evaluated via a two-sided Student’s *t* test. Horizontal lines represent the median, and individual dots represent distinct samples. (**D**) Boxplots of the Simpson index illustrating the relative clonal expansion in MG samples across CD8^+^ T subtypes, including CD8.Trm and CD8.Temra. Clonal expansion is indicated by a decrease in the Simpson diversity index. Differences between subtypes were evaluated using a Kruskal-Wallis test, with the resulting *P* values adjusted for multiple comparisons using the Bonferroni correction. (**E**) Significant up-regulation of *TNFSF13B* and *CHGB* in T_FH_ cells of patients with MG. Differentiation expression was performed on pseudobulk RNA-seq profiles (*n* = 6 MG, *n* = 3 NoMG), with an FDR < 0.05 considered significant. Heatmap visualization represents row *z*-scored CPM values. (**F**) Feature plot and dot plot validating the overexpression of *TNFSF13B* and *CHGB* in T_FH_ cells. Findings were corroborated using scRNA-seq data from the NU cohort and the Yasumizu *et al.* study (*3*), including both thymus and thymoma MG samples. Tcm, central memory T; Tem, effector memory T; Temra, effector memory T cells expressing CD45RA; T(agonist), agonist-selected T; CD8a/b(entry), early-entry CD8^+^ T; ILC, innate lymphoid cells.

Considering that MG is a T cell–dependent disease and our findings on the role of T cells in B cell interactions, we examined the T cell lineage in our integrated single-cell dataset. Using canonical cell type markers, we identified 24 distinct cell types within the non–B lymphocyte lineage (fig. S6, A to C). Among these, we observed large clusters of double-positive T cells, double-negative T cells, and proliferative T cells. Within the CD4^+^ T cell lineage, we identified multiple regulatory subtypes, including T follicular helper cells (T_FH_ cells), T_reg_ cells, and type 1 helper CD4^+^ effector memory T cells [CD4.Tem(Th1)]. When comparing patients with MG and NoMG patients, no T cell subtype was absent from either group (Fig. 3A). Proportional analysis revealed that the abundance of multiple regulatory subtypes, including T_FH_ cells, was comparable between MG and NoMG samples (Fig. 3B and fig. S6, D and E). Consistent with this, miloR differential abundance testing showed no statistically significant shifts for T_FH_ cells, although significant neighborhood-level shifts were detected in other regulatory subpopulations, such as T_reg_ cells.

In contrast to the clonotype results of B cells, our V(D)J sequencing of the T cell lineage revealed several clonotype expansions. The most expanded clonotype was clonotype337 (109 cells), followed by clonotype1695 (42 cells) and clonotype67104 (29 cells) (fig. S7A). When stratifying patients into MG and NoMG groups, the overall clonotype fractions were similar, although clonotype337 showed relatively greater expansion in patients with MG (fig. S7B). Furthermore, our repertoire diversity analysis, measuring richness and evenness, revealed no significant differences between MG and NoMG, as supported by various diversity metrics (Fig. 3C). This observation is consistent with the findings from Yasumizu *et al.* (*3*), indicating no substantial T cell clonotype expansion in MG pathogenesis. Further comparisons across T cell subtypes revealed significantly different between-subtype differences in clonotype expansion (Fig. 3D). Notably, CD8^+^ T cells, especially CD8.Trm and CD8.Temra, exhibited lower diversity. Analysis of the number of cells per clonotype within each subtype confirmed that expansions were predominantly found in CD8^+^ T cells, particularly CD8.Trm and CD8.Tem (fig. S7C). In contrast, CD4^+^ T cell subsets, including T_FH_ cells and T_reg_ cells, showed no evident clonotype expansions (fig. S7C).

In light of the significant role of T_FH_ cells in regulating B cell activity, we focused on T_FH_ cells and performed pseudobulk RNA-seq analysis comparing MG and NoMG groups. This analysis revealed nine significantly differentially expressed genes, with four up-regulated in the MG group (Fig. 3E). Notably, *TNFSF13B*, a molecule crucial for B cell maturation and antibody production, was significantly up-regulated in patients with MG. In addition, *CHGB*, which enables T_FH_ cells to produce and release high amounts of dopamine upon interaction with B cells, was also overexpressed (*14*). Dopamine has been reported to act as a signaling molecule that strengthens and accelerates T-B interactions, thereby enhancing GC output and antibody production—a previously unidentified mechanism of T_FH_ cell involvement in MG pathogenesis (*14*).

In addition to the pseudobulk RNA-seq analysis, we validated our findings using scRNA-seq data from both our cohort and public datasets. We confirmed the overexpression of *CHGB* not only in the thymus of patients with MG but also in thymoma with MG, as well as in the dataset from Yasumizu *et al.* (*3*) (Fig. 3F). Notably, *CHGB* overproduction was highly restricted to the T_FH_ cells, with little or no expression in other T cell subtypes. Furthermore, our scRNA-seq data also revealed increased *TNFSF13B* expression in T_FH_ cells, further validating the pseudobulk RNA-seq results.

### Altered T-B cell cross-talk revealed by tensor decomposition of cell-cell communication patterns

**Fig. 4.**
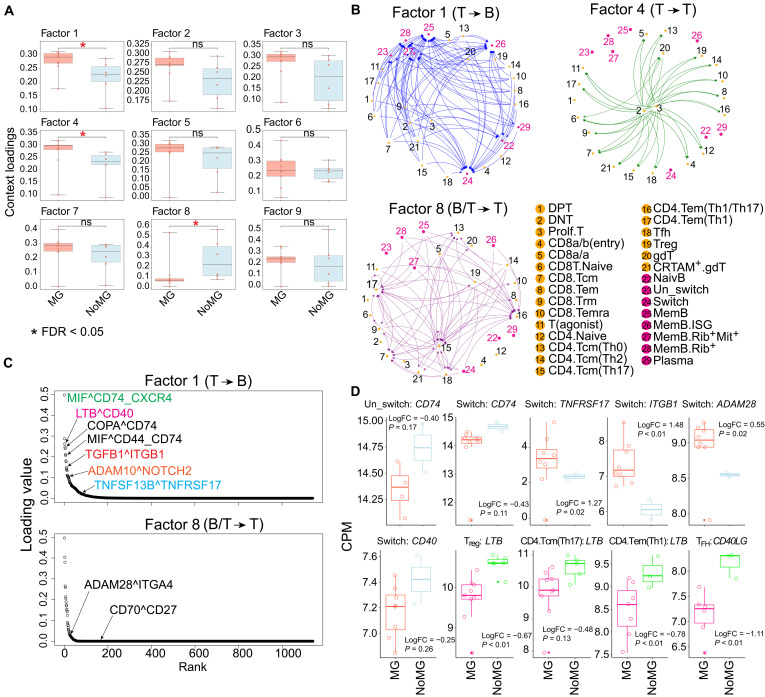
Differential cell-cell communication between MG and NoMG samples. (**A**) Boxplots illustrating the differences in context loadings between MG and NoMG samples for T-B cell-cell interactions. Factors 1, 4, and 8 were significantly different between MG (*n* = 9) and NoMG (*n* = 6). Intercellular context factorization was performed using the Tensor-Cell2cell framework within the LIANA+ package (v1.5.0); statistical significance was evaluated by the Mann-Whitney *U* test with FDR < 0.05. * indicates FDR < 0.05. (**B**) Network diagrams illustrating signal flow between cell types for the three significant factors. Factor 1 represents T-to-B communication; factor 4 represents T-to-T communication; and factor 8 represents B/T-to-T communication. (**C**) Scatterplots showing the top ligand-receptor pairs in factor 1 (differential T-to-B communication) and factor 8 (differential B/T-to-T communication). Ligand-receptor pairs are ranked by loading values in descending order. (**D**) Boxplots illustrating the expression of key ligand and receptor genes between MG and NoMG samples. Expression values (CPM) were obtained from pseudobulk RNA-seq profiles generated by aggregating raw single-cell counts per subject. Statistical significance was determined using the edgeR package. These data serve to validate the differential T-B interactions; therefore, raw *P* values and log_2_FC are reported. Each dot represents an individual subject [Switch: *n* = 8 MG, *n* = 2 NoMG*;* Un_switch: *n* = 4 MG, *n* = 2 NoMG; T_reg_ cells: *n* = 9 MG, *n* = 6 NoMG; CD4.Tcm(T_H_17): *n* = 9 MG, *n* = 5 NoMG; CD4.Tem (Th1): *n* = 7 MG, *n* = 3 NoMG; T_FH_ cells; *n* = 6 MG, *n* = 3 NoMG]*.* Whiskers represent 1.5× the interquartile range. ns, not significant.

Although differential gene expression analysis revealed several molecules crucial for MG pathogenesis, it could not fully capture the complex patterns of cell-cell interactions between disease states. Therefore, we performed tensor decomposition of cell-cell communication patterns between T and B cell subtypes to investigate differences in intercellular communication between MG and NoMG samples (*15*). This analysis identified three significant factors (factors 1, 4, and 8) associated with disease status (Fig. 4A and fig. S8A). Specifically, examination of signal flow between cell types showed that factor 1 represented T-B cell interactions, with signal flow from T cells to B cells; factor 4 represented T-T cell interactions; factor 8 represented interactions between B/T cells and T cells, with signal flow from B/T cells to T cells (Fig. 4B). Analysis of factor 1 revealed that the Switch B cells received strong signals from multiple T cell subtypes, including T_FH_ cells, CD4.Tcm(T_H_17), CD4.Tem(Th1/T_H_17), and CD4.Tcm(Th2).

To further explore the specific molecular mechanisms involved, we first analyzed the ligand-receptor pairs in factor 1. We observed alterations in antigen presentation activities, evidenced by the frequent involvement of *CD74*, a gene crucial for the major histocompatibility complex class II (MHC-II) antigen presentation pathway, among the top-ranked ligand-receptor pairs (Fig. 4C). Analysis of cell types with high *CD74* expression revealed that both Un_switch and Switch B cell subtypes showed the strongest expression (fig. S8B). However, when comparing MG and NoMG states within these two B cell subtypes, *CD74* expression was markedly reduced in patients with MG (Fig. 4D). This finding, together with our previous observation of reduced *CD1C* expression (Figs. 1C and 2F), strongly supports the notion that pathological B cell subtypes in MG have diminished antigen presentation capacity.

Another notable ligand-receptor pair involved the costimulatory molecule *CD40* (Fig. 4C), whose binding to its ligand enhances the antigen-presenting capacity of antigen presentation cells. Multiple CD4^+^ T cell subtypes, including T_reg_ cells, CD4.Tcm(T_H_17), and CD4.Tem(Th1), exhibited relatively lower expression of the ligand *LTB* in patients with MG. Correspondingly, Switch B cells in patients with MG showed lower expression of the *CD40* receptor (Fig. 4D). In addition to the LTB-CD40 pair, we also examined the well-known CD40L-CD40 interaction and found the reduced expression of *CD40LG* in T_FH_ cells (Fig. 4D). Expanding the analysis to a broader suite of genes within the MHC-II processing and presentation pathway revealed a coordinated down-regulation of the MHC-II molecule *HLA-DRA* and the antigen-processing protease *CTSS* in Switch B cells from MG samples (fig. S8C). Collectively, these findings consistently point toward a diminished molecular machinery for professional antigen presentation in this specific B cell subset.

In addition to reduced antigen presentation capacity, we identified molecular mechanisms that promote B cell activation and adaptation to the microenvironment. One such pathway is the *TNFSF13B* (BAFF)–*TNFRSF17* (BCMA) axis, whose interaction is essential for B cell long-term survival, differentiation, and antibody production. *TNFRSF17* expression was elevated in Switch B cells from patients with MG, in parallel with increased *TNFSF13B* production by T_FH_ cells (Figs. 3E and 4D). Since *TNFSF13B* is typically produced at high levels by innate immune cells such as monocytes, macrophages, and dendritic cells, we assessed its expression in these myeloid populations and likewise found higher levels in MG samples (fig. S8D). Together, these findings—reduced LTB/CD40L-CD40 signaling alongside increased BAFF-BCMA (TNFSF13B-TNFRSF17) signaling—suggest that Switch B cells transition from tightly regulated, T-dependent activation mediated by *CD40* to an innate-like, BAFF-driven survival program.

Switch B cells not only receive signals from T cells but also produce ligands that feed back to regulate T cell activity. Analysis of factor 8 revealed that Switch B cells overproduced *ADAM28*, which encodes ADAM metallopeptidase domain 28 and modulates T cell activation via the ADAM28-ITGA4 pathway (Fig. 4, C and D). As a molecule critical for promoting lymphocyte adhesion and transendothelial migration, *ADAM28* may contribute to T cell priming and enhanced adhesion, thereby facilitating the delivery of costimulatory signals necessary for full T cell activation.

### Spatial transcriptomics identifies Switch B cells within GCs

**Fig. 5.**
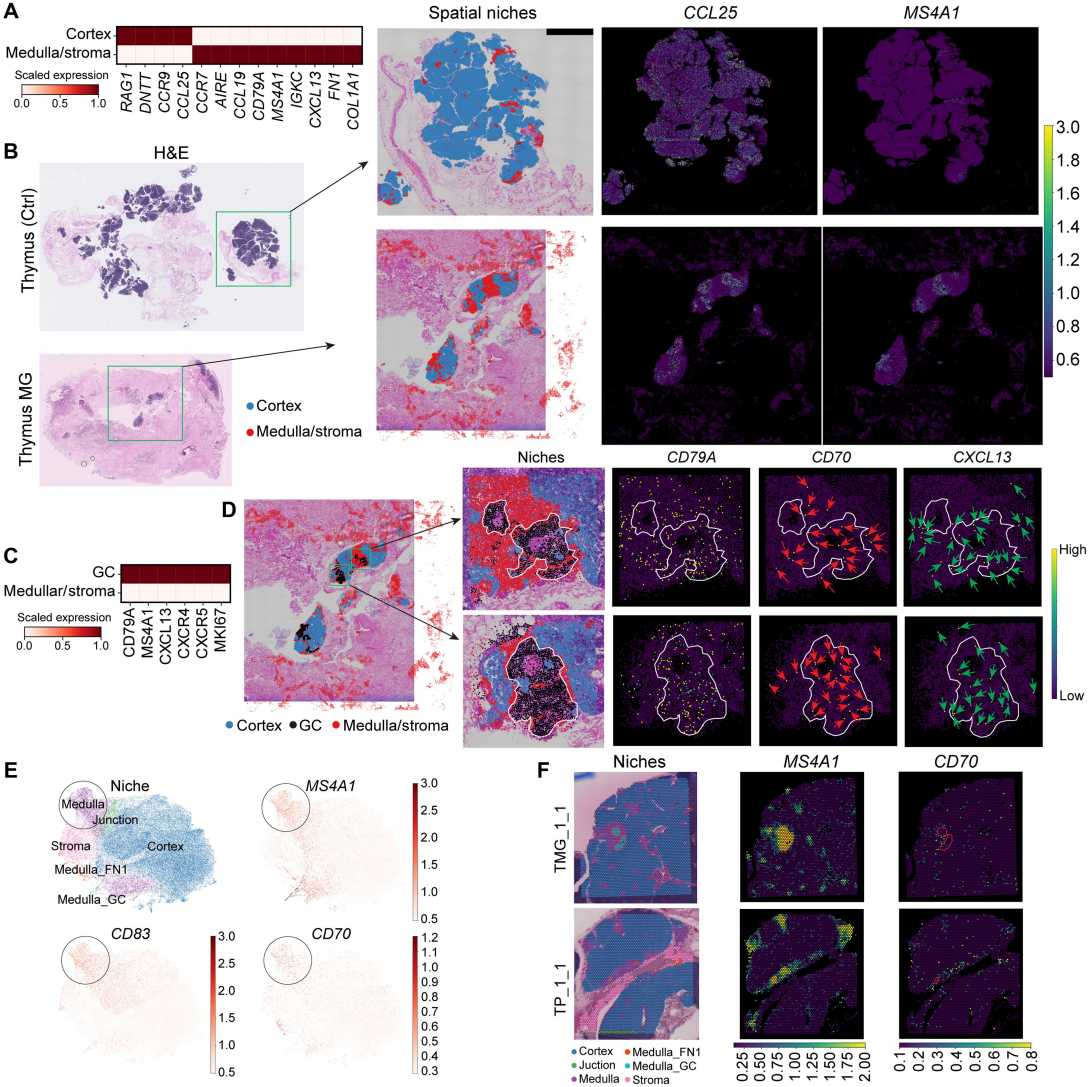
Pathological Switch B cells reside within ectopic GCs in the MG thymus. (**A**) Heatmap of marker genes defining the cortical and medullary/stromal niches in the NU cohort (Visium HD). Expression values are column-scaled (0 to 1) per gene to represent relative enrichment across niches rather than absolute abundance; lower values reflect lower relative expression within that niche and do not imply a complete absence of expression. (**B**) Spatial distribution of identified niches in thymus samples with and without MG. Corresponding hematoxylin and eosin (H&E) sections are shown with the Visium HD capture areas indicated by rectangles. Spatial niches were defined by local cell type composition (*k* = 60) and annotated using hallmark genes (e.g., *CCL25* for cortex). *MS4A1*^+^ B cells were primarily localized to the medullary niches and were specifically enriched in MG samples. (**C**) Heatmap of marker genes defining GC domains within the medullary niches. Expression values are column-scaled (0 to 1), representing relative gene enrichment across niches as described in (A). (**D**) Colocalization of Switch B cells within and surrounding GCs in a patient with MG, as evidenced by *CD70* expression. GCs are delineated by white boundaries. In the respective spatial plots, arrows highlight individual cells expressing *CD70* and *CXCL13* to facilitate visualization of their distribution within the GC niche. (**E**) UMAP projection of the medullary niches and Switch B cell localization from the Yasumizu *et al.* validation dataset (*2*). (**F**) Validation of Switch B cell localization within and around GCs in two independent MG samples (*2*). GC boundaries are indicated in red. Images shown are from the same samples subsequently analyzed in Fig. 6.

Pathological T-B cell interactions are frequently associated with the formation of organized lymphoid structures (*16*). Understanding these structures within the MG microenvironment—particularly those supporting class-switched B cell activity—is essential to defining disease pathogenesis. To investigate this, we performed high-resolution Visium HD spatial transcriptomics to dissect their spatial distribution and biological activities. Our niche analysis identified two main domains: cortex and medulla/stroma, each characterized by distinct niche-specific gene expression profiles (Fig. 5A). Notably, we observed a specific enrichment of B cells within the medulla/stroma niche.

Compared to NoMG controls, MG samples exhibited significant spatial aggregation of B cells within the medulla, quantified by Ripley’s L-statistics and consistent with the formation of GCs (Fig. 5B). We confirmed these structures as GCs through the localized overexpression of canonical markers (Fig. 5C). Spatial mapping further revealed that Switch B cells were mainly located within and surrounding these GCs in the MG sample (Fig. 5D and fig. S9), suggesting that GCs are a structural niche for their maturation and functional activity.

In addition to our in-house data, we cross-referenced Visium spatial transcriptomics from Yasumizu *et al.* (*2*). UMAP projection and spatial mapping of this independent cohort confirmed the localization of Switch B cells surrounding medullary GCs in patients with MG (Fig. 5, E and F), reinforcing the robustness of our observations.

### Spatial colocalization of key ligand-receptor pairs within and around the GCs

To investigate the aberrant ligand-receptor interactions that lead to the breakdown of immune tolerance in MG, we examined their spatial distribution within the thymic microenvironment using our spatial transcriptomic data. Cell type co-occurrence analysis revealed a high spatial enrichment of B cells alongside effector T cells and plasma cells (fig. S10A), with a patient with MG exhibiting increased communication frequency between B cells and both CD8^+^ and effector CD4^+^ T cell subsets.

**Fig. 6.**
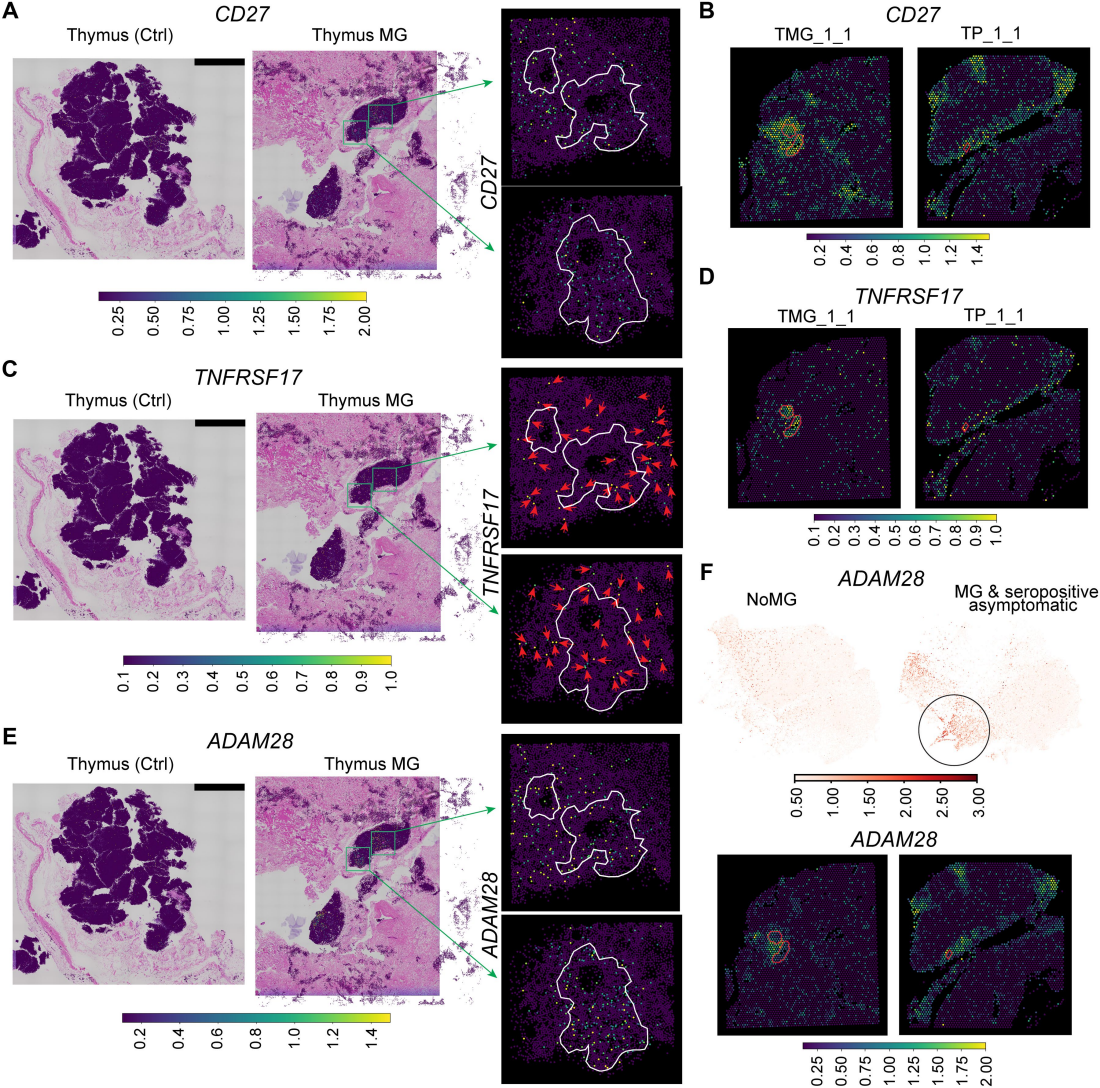
GCs as focal hubs for aberrant cell-cell communication in MG pathogenesis. (**A**, **C**, and **E**) Spatial expression of *CD27* (receptor for *CD70*), *TNFRSF17* (receptor for *TNFSF13B/BAFF*), and *ADAM28* within and surrounding GCs in the NU cohort (Visium HD). Insets provide magnified views of GC regions to resolve the spatial distribution of expressing cell types. Arrows in (C) highlight individual *TNFRSF17*^+^ cells to facilitate visualization within the GC microenvironment. (**B** and **D**) Validation of *CD27* and *TNFRSF17* spatial localization within and around GCs of MG samples using the Yasumizu *et al.* Visium dataset (*2*). (**F**) UMAP projection demonstrating the enriched expression of *ADAM28* within the Medulla_GC niche of MG samples. Spatial plots of two independent MG samples from the Yasumizu *et al.* study (*2*) confirm the localization of *ADAM28*^+^ cells within and proximal to GCs. Images shown are from the same samples presented in Fig. 5 to illustrate gene spatial distribution.

Quantitative proximity analysis confirmed the physical clustering of B and T cells within and around medullary GCs in MG (fig. S10B), providing a structural validation of the interactome predicted by our scRNA-seq data. To validate specific signaling axes, we examined the *CD70-CD27* axis; *CD27* expression consistently colocalized with GCs across both our Visium HD and external spatial datasets (Fig. 6, A and B). Furthermore, we investigated the spatial expression of *TNFRSF17* (BCMA), a receptor up-regulated by Switch B cells to promote long-term survival via interactions with T_FH_ cells (Fig. 4, C and D). Our results demonstrate that the GCs provide the specific microenvironment necessary for *TNFRSF17* overexpression, thereby mediating Switch B cell persistence and activity (Fig. 6, C and D). In addition, our spatial transcriptomics confirmed the enrichment of *ADAM28*—a metalloproteinase overexpressed by Switch B cells for T cell interaction—within these GC niches (Fig. 6, E and F, and fig. S9). Together, these results indicate that GCs serve as specialized structural niches that support the survival and functional reprogramming of pathological B cells, potentially contributing to the breakdown of immune tolerance in MG pathogenesis.

## DISCUSSION

This study suggests a fundamental shift in myasthenia gravis pathogenesis. Rather than resulting from simple autoantigen exposure or clonal expansion of autoreactive B cells, MG arises from a reprogramming of immune tolerance checkpoints within the thymus. We suggest that pathological B cells escape normal selection pressures by switching from classical T cell–dependent activation to an innate survival program driven by BAFF signaling (*17*–*19*). This tolerance checkpoint swap represents a potentially new paradigm for understanding not only MG but potentially other autoimmune diseases where clinical severity disconnects from autoantibody levels (*16*, *20*).

Our data suggest that class-switched B cells that acquire unique survival advantages within thymic GCs represent a key mechanism that drives autoimmunity. These cells exhibit three critical features that distinguish them from normal B cells. First, they show markedly reduced antigen presentation capacity through down-regulation of MHC-II genes including *CD74* and *CD1C*. Second, they demonstrate diminished *CD40* expression and reduced responsiveness to *CD40L* from T cells. Third, they compensate for these deficits by up-regulating *TNFRSF17* (*BCMA*) to engage BAFF survival signals. This molecular reprogramming allows B cells to survive without stringent antigen-specific selection. The polyclonal nature of the B cell repertoire we observed supports this model. Rather than clonal expansion of high-affinity autoreactive cells, MG involves the survival of diverse B cell populations that would normally be eliminated through tolerance checkpoints.

This mechanism offers a potential resolution to longstanding clinical paradoxes, for example, the discordance between antibody titers and disease severity. Antibody levels measure only the output of plasma cells, while disease activity potentially reflects the strength of survival signals maintaining the entire autoreactive B cell pool. Patients with high survival tone in their thymic niches may experience severe disease even with modest antibody titers. Conversely, those with lower survival signaling may have high titers but milder symptoms. The variable efficacy of thymectomy also becomes explicable. Class-switched B cells that depend on *BAFF* rather than antigen-specific signals can seed peripheral sites before surgery, as suggested by our data of tissue and blood B cells. These cells can establish extrathymic reservoirs that persist after thymic removal. Our identification of the *ADAM28-ITGA4* axis suggests that B cells acquire tissue remodeling and adhesion programs—evidenced by *ADAM28* overexpression within ectopic GCs—before thymic exit. These programs likely facilitate the seeding of extra-thymic reservoirs, such as the bone marrow or lymph nodes, where autoreactive clones can persist long after the thymus is removed. While longitudinal studies and peripheral tissue biopsies are required to map these peripheral sites definitively, our data highlight this axis as a possible therapeutic target for addressing both thymic and systemic pathogenic B cell persistence

The identification of *BAFF* as a central driver has broader implications for autoimmunity. *BAFF* overproduction occurs in multiple autoimmune diseases including systemic lupus erythematosus and rheumatoid arthritis (*21*). Our findings suggest that *BAFF* may enable a common mechanism whereby autoreactive B cells circumvent normal tolerance checkpoints. The parallel up-regulation of *CHGB* in T_FH_ cells provides additional insight. This molecule enables dopamine release at immune synapses, accelerating B cell activation independent of antigen affinity (*14*). Together, BAFF and dopamine signaling create a permissive environment where the quantity of survival signals overrides the quality of antigen recognition. This framework may explain why *BAFF* inhibition shows efficacy across diverse autoimmune conditions despite different target antigens. Our findings identify multiple potential therapeutic opportunities beyond current approaches. First, measuring serum levels of soluble BAFF and BCMA by-products could provide better biomarkers than antibody titers for monitoring disease activity and predicting treatment response as these markers directly reflect the survival tone driving pathogenic B cells. Second, the BAFF-BCMA axis represents an actionable target. Belimumab, a Food and Drug Administration–approved BAFF inhibitor, warrants investigation in MG clinical trials. *BCMA-*targeted therapies under development for multiple myeloma could be repurposed for severe MG cases. Third, restoring CD40 signaling through CD40 agonists might reestablish normal selection checkpoints. This approach could eliminate low-affinity autoreactive cells while preserving protective immunity. Fourth, blocking *ADAM28-*mediated adhesion could prevent B cells from establishing peripheral reservoirs. Last, combination strategies targeting both survival signals and adhesion programs may prove more effective than single agent approaches.

The spatial architecture we identified points toward novel therapeutic vulnerabilities. By resolving the spatial organization of these pathological niches, we show that GCs in the MG thymus act as privileged zones that concentrate survival signaling. Disrupting these structures pharmacologically could eliminate the protective microenvironment sustaining autoreactive cells. The colocalization of Switch B cells with specific T cell subsets further suggests that modulating these localized interaction zones—rather than broad systemic immunosuppression—may offer a more precise therapeutic approach for MG.

This study has certain limitations. First, the cross-sectional design prevents us from determining whether the tolerance checkpoint swap initiates disease or develops secondarily. Longitudinal studies examining thymic changes from disease onset through progression will be necessary to establish causality. Second, our small sample size restricted the inclusion of multiple covariates (e.g., sex and age) in the pseudobulk RNA-seq models without risking overfitting. While a sensitivity analysis for this specific analysis confirmed that the direction of effect (log fold change) for *CD1C* and *LILRA4* remained consistent, the increased model complexity resulted in a reduction in statistical power. Furthermore, we acknowledge the immunopathological differences between thymoma-associated MG and thymic hyperplasia. While both result in pathogenic autoantibody production, thymoma-associated MG involves impaired negative selection within a neoplastic microenvironment, whereas nonthymomatous MG often features ectopic GC formation. Although we included these conditions as a covariate where possible, our sample size precluded sufficient statistical power for this adjustment in all sub-analyses. Separately, we acknowledge that certain cell type–specific observations (e.g., Figs. 1C, 2E, 3, C and D, and 4D) are constrained by the limited total number of available patients and the inherent rarity of specific cell subsets. Consequently, these comparisons may be interpreted as exploratory, and future studies with larger cohorts are required to validate these findings. Moreover, our cohort limits the ability to detect differences between antibody subtypes (e.g., MuSK versus LRP4), and while our data suggest a role for BAFF and altered antigen presentation in Switch B cells, these findings require direct validation through serum protein measurements and ex vivo coculture functional assays.

In conclusion, we have identified a tolerance checkpoint swap that provides previously unknown insights into MG pathogenesis. This shift from antigen-dependent selection to BAFF-driven survival offers a mechanistic explanation for longstanding clinical paradoxes in MG and identifies promising molecular targets for therapeutic intervention. By restoring the balance between survival signals and selection checkpoints, it may be possible to reestablish immune tolerance without the need for broad immunosuppression. Hence, these findings provide a framework for improving treatment strategies in MG and potentially other autoimmune diseases driven by similar pathogenic mechanisms.

## MATERIALS AND METHODS

### Human sample preparation and single-cell sequencing

Human tissue samples were obtained from Northwestern Memorial Hospital in Chicago, IL. All research protocols were approved by the Institutional Review Board (IRB #STU00216259) of NU, and written informed consent was obtained from all participants before tissue collection. Two library preparation methods were used: 10x Genomics Chromium Single Cell 3′ v3 chemistry and Single Cell 5′ chemistry with V(D)J sequencing. Four samples were prepared using the 3′ v3 method at the Metabolomics Core Facility at NU. Tissues were dissociated into single-cell suspensions and sorted via flow cytometry to enrich for epithelial cells (CD45^−^EPCAM^+^), immune cells (CD45^+^), and other cells (CD45^−^EPCAM^−^). Each compartment was then subjected to single-cell sequencing. The remaining 19 samples were processed with the Chromium Single Cell 5′ chemistry with V(D)J sequencing at the NuSeq Core at NU. Similar dissociation and sorting procedures were applied, but the three compartments were recombined for each sample in varying proportions to prevent overrepresentation of immune cells and ensure balanced representation across cell types. For both methods, sequencing was performed on an Illumina platform, and raw data were demultiplexed with Cell Ranger (v6.0.0 for 3′ v3 and v7.1.0 for 5′ chemistry) using standard protocols. Sequencing reads were aligned to the human reference genome (GRCh38) to generate raw gene expression counts per cell.

### Single-cell data quality control and integration

scRNA-seq data were first processed with the SoupX toolkit (v1.6.2) to remove ambient RNA contamination (*22*), followed by doublet detection and removal using scDblFinder (v1.18.0) (*23*). Additional quality control was performed with Seurat (v4.3.0) (*24*); cells were excluded if they had fewer than 200 or more than 7500 detected genes, fewer than 400 or more than 40,000 UMI counts, or greater than 10% mitochondrial gene content. To increase statistical power, we also incorporated two publicly available datasets: thymoma with MG samples from Yasumizu *et al.* and thymic epithelial tumor samples from Xin *et al.* (table S2) (*3*, *6*). For the Xin *et al.* cohort, raw FASTQ files were downloaded and aligned to the GRCh38 reference genome using Cell Ranger, followed by the same quality control (QC) pipeline applied to our in-house data. For scRNA-seq data from Yasumizu *et al.* study, we downloaded the count matrix from the Single Cell Portal and retained only the cells that had passed the original authors’ quality filters for further analysis.

Raw count matrices from all samples were integrated using scvi-tools (*7*), a deep probabilistic framework for single-cell omic analysis that leverages GPU acceleration with mini-batching to efficiently model complex datasets while correcting for technical variation. Each sample was treated as a distinct batch, and the top 3000 highly variable genes were selected to support dimensionality reduction and integration. The integrated latent space was computed using UMAP based on scVI-inferred embeddings and used for downstream analyses. Integration quality was evaluated by examining cell distribution across datasets within clusters, variation in gene and UMI counts among clusters, and separation of distinct cell populations. We further applied the scIB package to compute evaluation metrics (*8*): The silhouette score was used to assess batch correction by measuring how well cells from the same batch clustered relative to cells from different batches, while NMI was used to assess biological conservation by comparing clustering results with annotated cell labels.

### Cell type annotation

We first classified cells into structural (epithelial, endothelial, and stromal) and immune compartments using compartment-specific gene markers (*EPCAM*, *PECAM1*, and *PTPRC*) and then extracted cells from each compartment for fine-resolution subclustering in Seurat. Cell type identity was determined through manual curation of cluster-specific markers, with results further validated and refined using CellTypist (v1.6.3) (*25*). For the CellTypist analysis, we applied the well-annotated reference labels from a previous study as well as the Immune V2 dataset from the Cell Type Encyclopedia (*3*, *26*). For manual annotation, we conducted differential gene expression analysis between clusters to identify cluster-specific markers, iteratively adjusting the clustering resolution to best capture cell type distinctions and align with known populations. During annotation, clusters containing doublets or low-quality cells were removed. Where possible, our annotations were further cross-validated against the original published datasets to ensure consistency and robustness.

### Pseudobulk RNA-seq analysis

For the pseudobulk RNA-seq analysis, we extracted the relevant cell populations from the integrated object and summed the raw counts of all cells per sample to enable patient-level analysis. Only samples with cell numbers above the threshold (e.g., *n* = 25 for B cell pseudobulk RNA-seq) were retained. Specifically, B cell analysis was performed on aggregated B and Prolif.B clusters, excluding plasma cells, while all epithelial subsets were pooled for the epithelial cell analysis. Differential gene expression analysis was performed using the edgeR package (v4.2.2) *(**27**)*, restricting the analysis to protein-coding genes. A linear model accounting for disease status and sequencing chemistry was fitted where sample sizes permitted; otherwise, disease status was used as the sole covariate. Post hoc comparisons were then performed to identify differential expression between MG and NoMG samples. FDRs were estimated using the Benjamini-Hochberg procedure. To evaluate the potential confounding effects of patient demographics in the B cell pseudobulk analysis, a sensitivity analysis was performed by including sex and age as additional covariates. Genes with FDR < 0.05 and an absolute log_2_ fold change > 1 were considered statistically significant in the primary analysis. GSEA was performed using the MSigDB Reactome database (v7.5.1) as the reference (*28*), implemented with the fgsea package (v1.30.0). Pathways with FDR < 0.05 were considered statistically significant.

### Cell subtype proportion analysis

To assess cell type distribution, we calculated the proportion of each cell subtype within its parent cell lineage for every sample. Within the B cell lineage, the relative abundance of each B cell subtype per sample was used for analysis; patients with insufficient cell counts (<50 cells) and subtypes with low total abundance (<200 total cells) were excluded to ensure robust estimates. To maintain consistency, this proportion analysis was restricted to tissue data from the NU cohort. Differential abundance between MG and NoMG conditions was quantified using miloR (v2.0.0) (*13*). For the T cell lineage, we subsampled overrepresented subtypes to a maximum of 4000 cells to balance the K-nearest neighbor graph, which was constructed using *k* = 20 and *d* = 30. We adjusted the proportion in makeNhoods to ensure an average neighborhood size over 5 × *N*_samples_. We then assigned cell type labels to each neighborhood based on the majority population; neighborhoods with a cell type fraction below 0.7 were excluded to prevent cross-lineage contamination. Differential abundance was visualized using beeswarm plots. Because of limited sample size, no covariates were included in the B cell composition analysis; however, for T cell subsets, thymic hyperplasia and thymoma status was incorporated into the design matrix to account for pathological variation.

### RNA velocity analysis

To analyze the developmental dynamics of B cell subsets, we performed RNA velocity analysis using the scVelo (v0.2.4) package (*29*, *30*). Spliced and unspliced mRNA abundances were quantified via the velocyto pipeline and integrated with our thymic cohorts. Following the selection of 5000 highly variable genes and filtering for a minimum of 20 shared counts, we computed first- and second-order moments using the top 30 principal components with *k* = 80 neighbors. We then used the dynamical model to estimate gene-specific kinetic parameters, including transcription, splicing, and degradation rates. Velocity vectors were projected onto the UMAP embedding, and transitions between B cell states were visualized using velocity_embedding_grid to identify the developmental trajectories of pathological B cell subsets in MG.

### Bioinformatic analysis of V(D)J sequencing data

For the B cell receptor/T cell receptor (TCR) sequencing data, we used the djvdj (v0.1.0) package, including only cells with high-quality transcriptomic data. Samples with fewer than 10 cells were excluded to ensure robustness. Clonotypes were defined on the basis of CDR3 amino acid sequences, and only clonotypes with exactly two chains were retained for further analysis. Cells without an assigned clonotype were removed. The final dataset for the B cell lineage included 5061 cells (1225 from NoMG patients and 3836 from patients with MG). For the TCR data, 19,602 cells were analyzed (4917 from NoMG patients and 14,685 from patients with MG). Repertoire diversity was estimated at the individual patient level by calculating the Shannon, Simpson, and Pielou’s evenness indices from each patient’s complete clonotype abundance profile using formulas from the abdiv package. Statistical comparisons between two groups (e.g., MG versus NoMG) were evaluated using a two-sided Student’s *t* test. For comparisons involving more than two groups (e.g., distinct T cell subtypes), a Kruskal-Wallis test was applied, with *P* values adjusted for multiple testing using the Bonferroni correction. A *P* value less than 0.05 was considered statistically significant.

### Differential cell-cell communication between MG and NoMG samples

Differential cell-cell communication analysis was performed using the LIANA+ package (v1.5.0) (*31*), with the Tensor-Cell2cell analytical framework applied for intercellular context factorization (*15*). Specifically, we extracted B and T cell subtypes from the tissue data of the NU cohort in our integrated object and excluded cell types with fewer than 200 cells from further analysis. We first inferred consensus-based rankings of cell-cell interactions for each individual sample, followed by tensor decomposition to investigate the factors and their associated loadings. The weights of ligand-receptor interactions contributing to each factor were then ranked for subsequent analysis. To identify factors significantly associated with MG, we compared context loadings between MG and NoMG samples using the Mann-Whitney *U* test, with an FDR < 0.05 considered statistically significant.

### Visium HD spatial transcriptomic data analysis

We selected three representative patients—from a thymus without MG, a thymus with MG, and a thymoma with MG—for Visium HD spatial transcriptomic analysis. Formalin-fixed, paraffin-embedded specimens were sectioned at 5 μm and processed at the NUSeq Core following the manufacturer’s protocol. Images were manually aligned using Loupe Browser (v8.0.0), and genome mapping was performed with Space Ranger (v3.1.0; 10x Genomics) against the human reference genome (GRCh38). To reconstruct single-cell–level like resolution, the Bin2cell package (v0.3.3) was used to aggregate 2-μm bins based on hematoxylin and eosin morphology and gene expression, at a resolution of 0.5 μm per pixel (*32*). These constructed single-cell–like objects (hereafter “cells”) were used for downstream analyses, which were conducted with Scanpy (v1.10.3) (*33*). In this study, we excluded cells with fewer than 80 or more than 40,000 features or with mitochondrial content greater than 20%. Cell types were inferred using CellTypist with a confidence score threshold of 0.7, by referencing our scRNA-seq dataset to ensure consistent annotations (*25*). UMAP projections were generated using the BANKSY (v1.3.4) to incorporate spatial context (lambda = 0.2, neighborhood size = 15) (*34*).

To evaluate the spatial distribution of B cells in the MG sample, we calculated Ripley’s L-statistics via the Squidpy package (v1.6.1) against a null distribution of complete spatial randomness (100 permutations) (*35*). To characterize the local spatial architecture, we performed neighborhood enrichment analysis to quantify the colocalization preferences between cell type pairs within the thymic microenvironment. To identify spatial niches, we calculated local cell type composition vectors within the 60 nearest neighbors of each reconstructed cell and used a shared nearest neighbor algorithm to delineate tissue domains. These niches were further annotated according to their hallmark markers, such as the overexpression of *CCL25* in the cortex and *CCL19* in the medulla. To identify GCs, we focused on the medulla/stromal niche and applied CellCharter (v0.3.3) to delineate the spatial domains characteristic of these structures (*36*).

To quantitatively assess spatial organization relative to GCs, we computed the minimum Euclidean distance from each cell to the nearest GC-designated cell. This was implemented using the NearestNeighbors algorithm (scikit-learn v1.6.1), where a nearest-neighbor index was constructed from GC cell coordinates (*n* = 1) to calculate the shortest spatial distance (dist_to_GC). Distances were measured in spatial coordinate units (pixels).
