# Supplementary material for: Myasthenia thymus reprograms class-switched B cells into BAFF-dependent survivors
Source: Sci Adv. 2026 Aug 12;12(33):eaec3842. doi: 10.1126/sciadv.aec3842 (PMC13464467; doi:10.1126/sciadv.aec3842)
Supplement: Supplementary file 1 [file sciadv.aec3842_sm.pdf]

Science Advances
AAAS

Supplementary Materials for

Myasthenia thymus reprograms class-switched B cells into BAFF-dependent survivors

Yuanqing Yan *et al.*

Corresponding author: Ankit Bharat, ankit.bharat@nm.org

Sci. Adv. **12**, eaec3842 (2026)
DOI: 10.1126/sciadv.aec3842

This PDF file includes:

Figs. S1 to S10
Tables S1 and S2

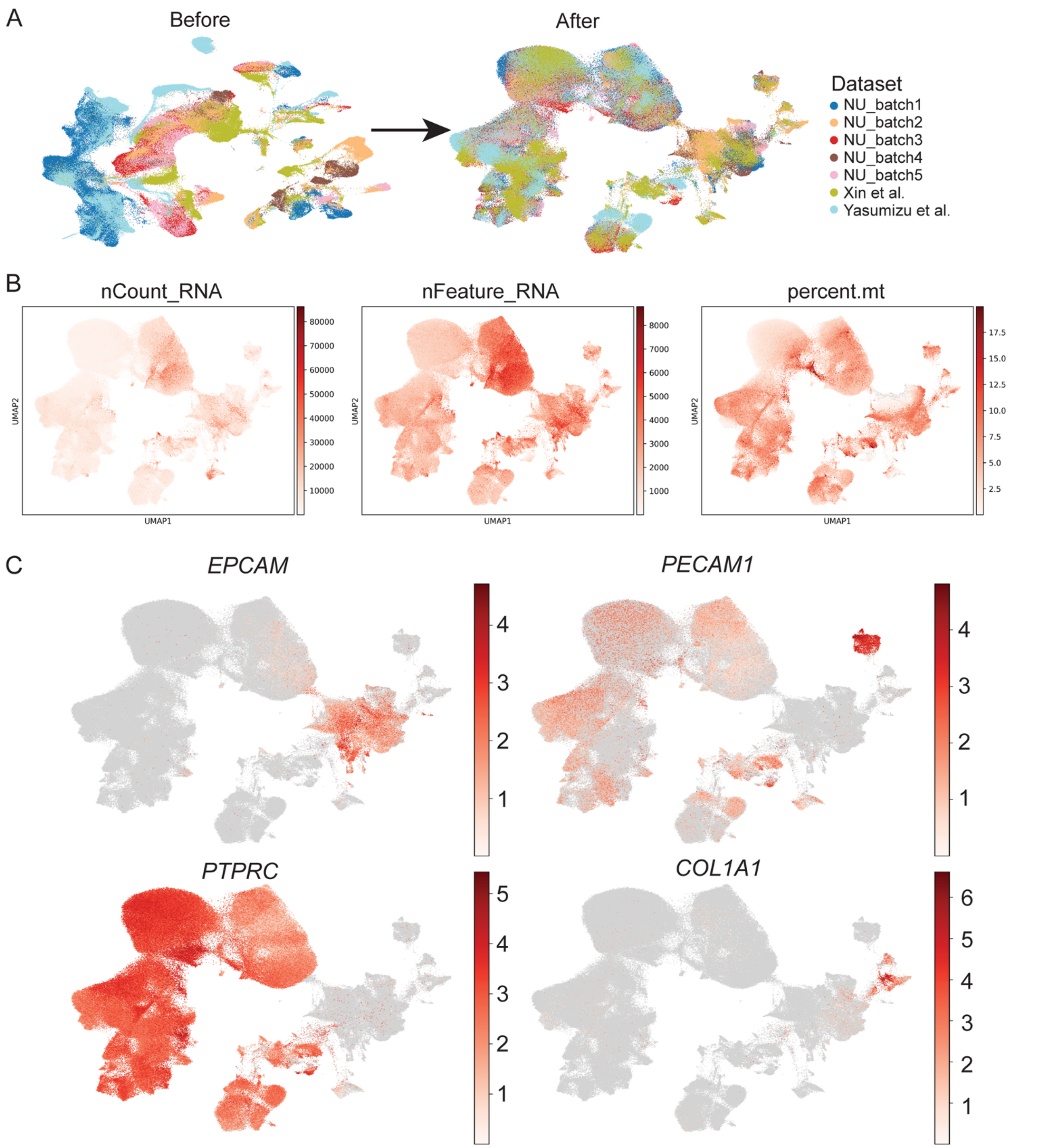

Figure S1. Quality control and integration of single-cell transcriptomic data. (A) UMAP projections illustrating cell clustering before (left) and after (right) batch effect correction using scvi-tools (v1.1.6.post2). The harmonization of cells across different batches indicates robust data integration. (B) Quality control metrics across cell clusters, including the number of detected genes (nFeature_RNA), total UMI counts (nCount_RNA), and the percentage of mitochondrial gene expression (percent.mt). (C) Identification of the four primary cellular

compartments (epithelial, endothelial, stromal, and immune) based on the canonical markers *EPCAM, PECAM1*, and *PTPRC* (CD45).

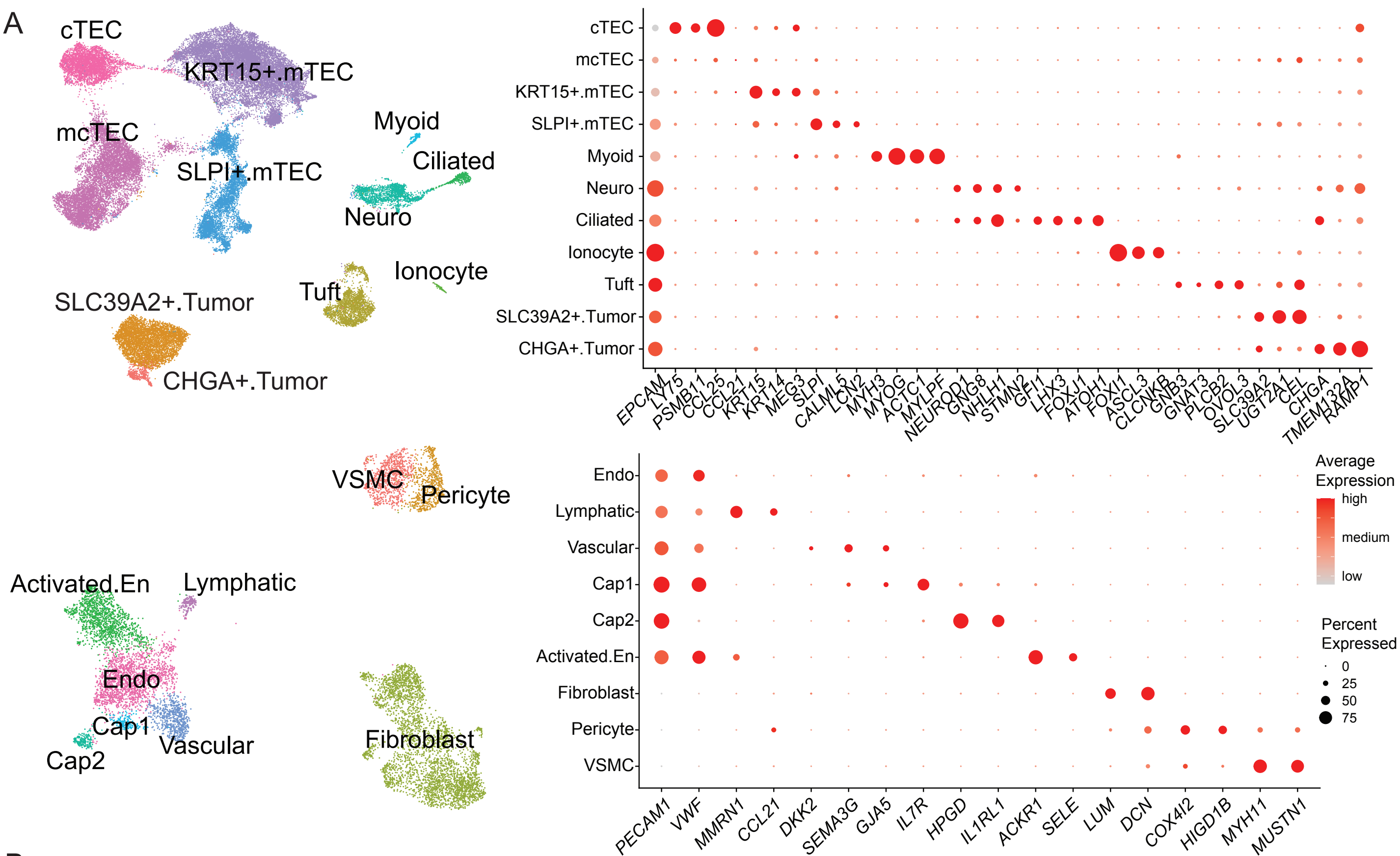

B

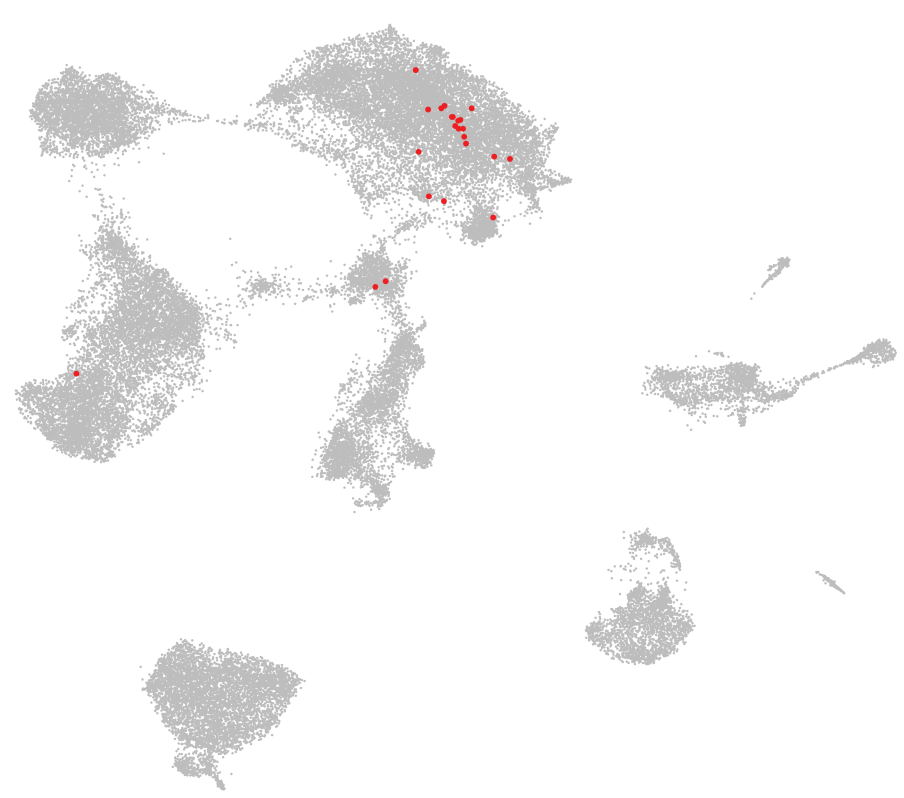

C

Gene	LogFC	FDR
CHRNA1	1.286	0.636
CHRNA3	-1.389	0.458
CHRNA4	0.438	0.870
CHRNA5	-0.258	0.840
CHRNA7	1.310	0.289
CHRNA9	1.498	0.503
CHRNA10	1.387	0.424

Gene	LogFC	FDR
CHRNE	0.751	0.507
CHRND	2.635	0.217
CHRNG	2.107	0.311
CHRNB1	0.365	0.734
CHRNB2	0.688	0.759
CHRNB4	-1.771	0.371

Figure S2. Identification of structural cell types and *AChR* gene expression in epithelial cells. (A) UMAP visualization (left) and dot plot (right) illustrating the identification of different cell types within structural cell compartment. Abbreviations: TEC: thymic epithelial cells; cTEC: cortical TEC; KRT15+.mTEC: KRT15-expressing medullary TEC; SLPI+.mTEC: SLPI-expressing medullary TEC; Myoid: thymic myoid cells; mcTEC: thymic epithelial cells with intermediate levels of cTEC and mTEC markers; Neuro: neuroendocrine cells; SLC39A2+.Tumor: SLC39A2-expressing thymic tumor cells; CHGA+.Tumor: CHGA-expressing thymic tumor cells; VSMC: vascular smooth muscle cells; Endo: endothelial cells; Activated.En: activated endothelial cells;

Cap: capillaries; Lymphatic: lymphatic endothelial cells. (B) Feature plot showing the embedding of neuromuscular thymic epithelial cells (nmTECs) within the KRT15+ mTEC cluster. nmTEC identity was assigned by cross-referencing cell barcodes with those reported in the Yasumizu et al. (2022) study (*3*). (C) Differential expression analysis of *AChR* genes in epithelial cells comparing MG and NoMG samples. Pseudo-bulk profiles were generated by aggregating scRNA-seq counts per sample. Statistical significance was determined using a linear model in edgeR (v4.2.2) package, adjusting for disease status and sequencing chemistry. P-values were adjusted for multiplicity using the Benjamini–Hochberg procedure, with an FDR < 0.05 considered statistically significant.

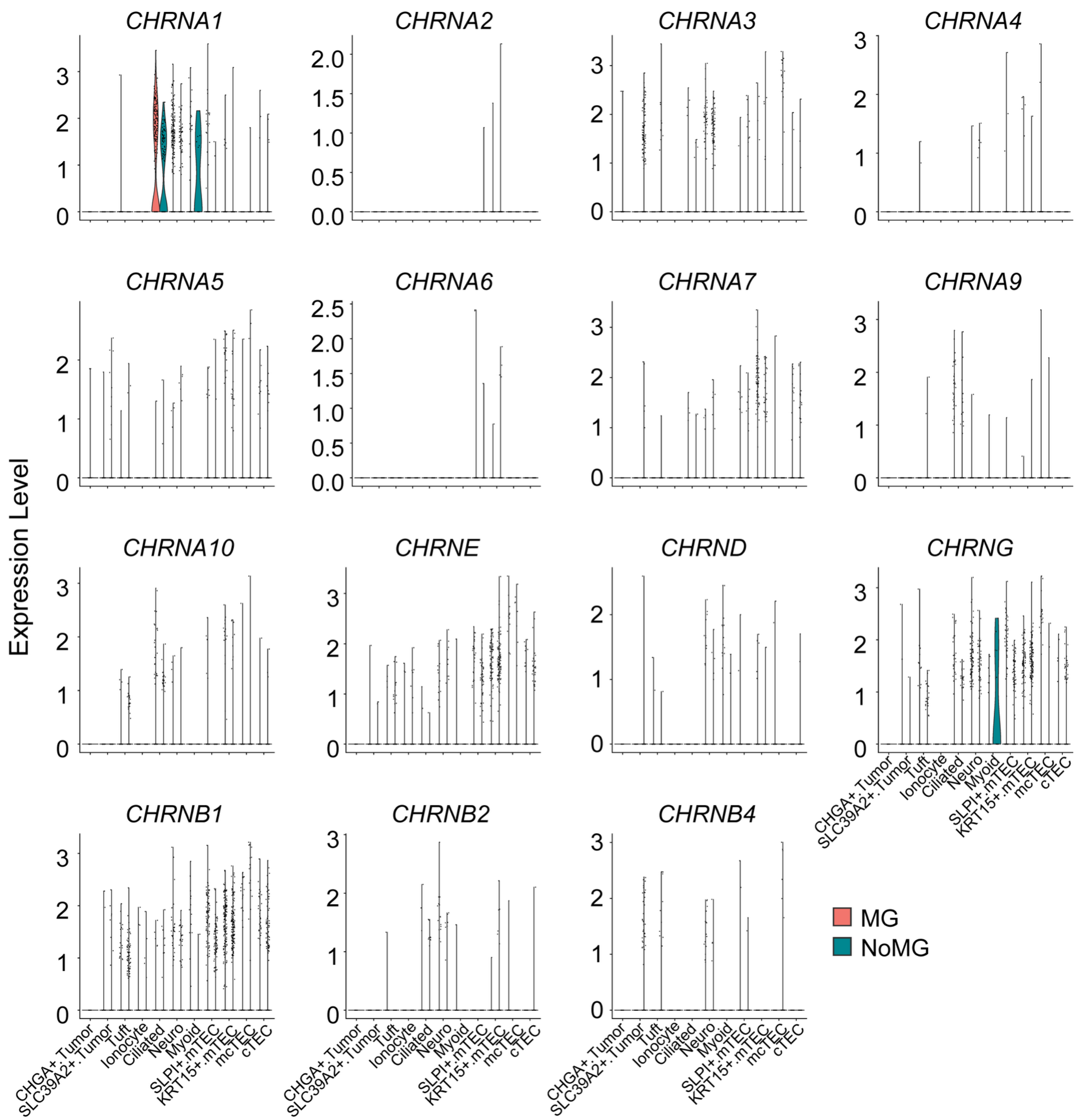

Figure S3. Expression of *AChR* genes across thymic epithelial cell subsets. Violin plots depicting the expression levels of *AChR* subunits across various cell types within the epithelial compartment in MG and NoMG samples.

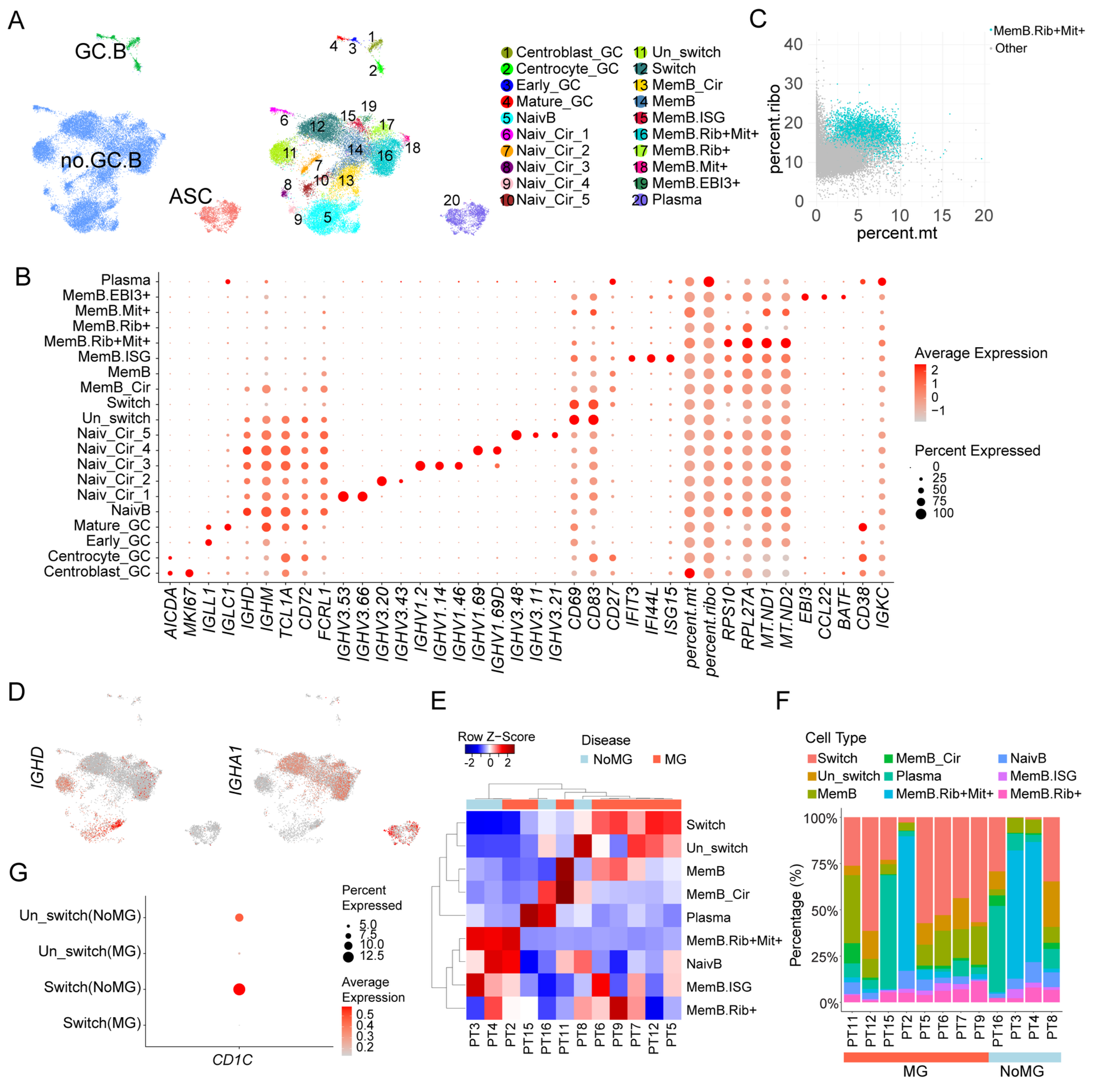

Figure S4. Identification and characterization of B-cell subsets. (A) UMAP visualization of B-cell populations, categorized into three major lineages (germinal center B cells (GC.B), non-germinal center B cells (no.GC.B), and antibody-secreting cells (ASCs)), and 20 refined sub-clusters. Abbreviations: NaivB: naïve B cells; Naiv_cir_1: naïve circulating B cells; MemB: memory B cells; MemB_Cir: circulating memory B cells; MemB.Rib+Mit+: memory B cells with high ribosomal and mitochondrial gene content; MemB.EBI3+: EBI3-expressing memory B cells; MemB.ISG: interferon-stimulated gene-expressing memory B cells. (B) Dot plot illustrating the expression of marker genes used to identify the different B-cell subsets. (C) Scatter plot

demonstrating the identification of specific B-cell subsets (MemB.Rib+, MemB.Mit+, MemB.Rib+Mit+) based on mitochondrial and ribosomal gene percentages. (D) Feature plots showing *IGHD* expression in Un_switch B cells and *IGHA1* expression in the Switch B-cell subset. (E) Heatmap illustrating the proportional distribution of B-cell subtypes across patients. Hierarchical clustering of patients (columns) was performed using the complete linkage method. Data represent the percentage of total B cells per patient, normalized via row Z-score scaling to compare MG and NoMG groups. (F) Stacked bar plot showing the raw cellular composition per patient, complementary to panel E but without row scaling. (G) Dot plot showing *CD1C* expression in Switch and Un_switch B-cell subsets. A reduced expression pattern of *CD1C* was observed in both Switch and Un_switch B cells from MG samples compared to NoMG controls.

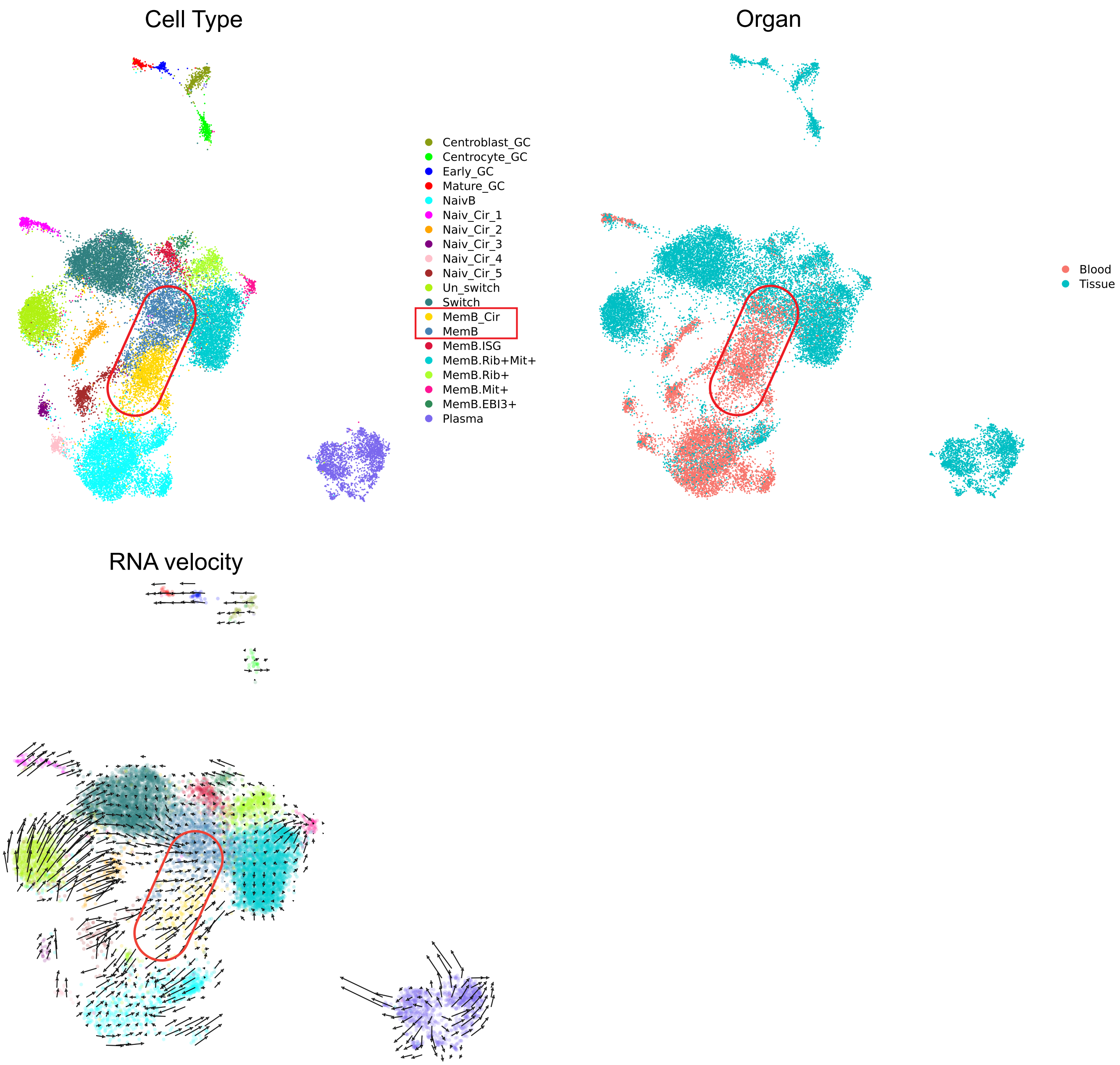

Figure S5. Coexistence of circulating and tissue-resident memory B cells in MG. UMAP visualization highlighting the coexistence of circulating and tissue-resident memory B-cell populations (red ellipse). RNA velocity analysis of the NU cohort illustrating the developmental trajectory of the Switch B-cell subtype. The velocity vectors indicate that Switch B cells likely serve as the precursors to the memory B-cell pool in the MG thymus.

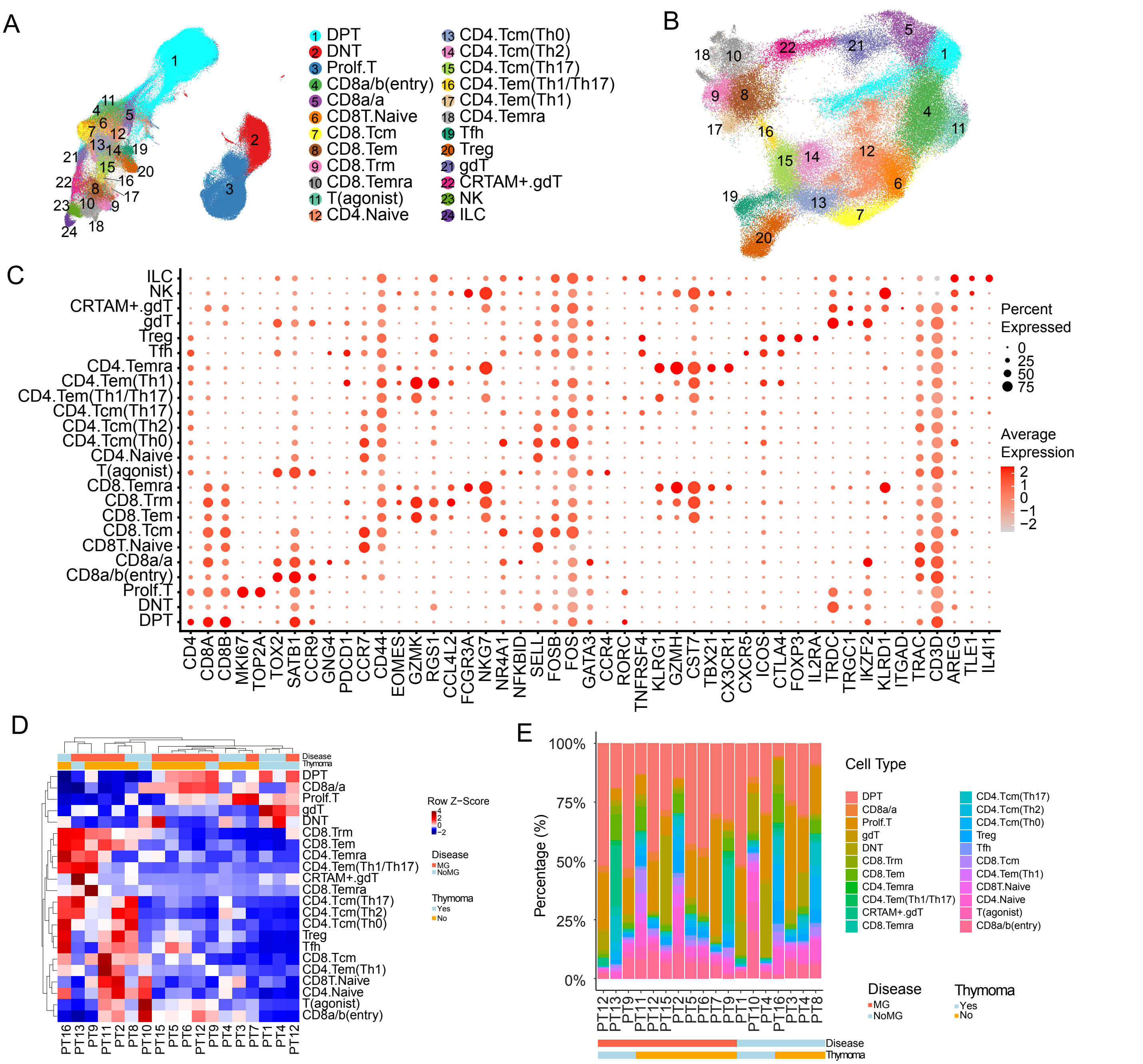

Figure S6. Identification and characterization of T-cell subsets. (A) UMAP visualization of the 24 T cell subtypes identified in this study. Abbreviations: DPT: double-positive T cells (CD4+CD8+); DNT: double-negative T cells (CD4-CD8-); Treg: regulatory T cells; Tfh: follicular helper T cells; Tcm: central memory T cells; Tem: effector memory T cells; Temra: effector memory T cells expressing CD45RA; gdT: gamma-delta T cells; T(agonist): agonist-selected T cells; CD8a/b(entry): early-entry CD8+ T cells; Prolf.T: proliferating T cells; ILC: innate lymphoid cells. (B) Refined UMAP visualization following the removal of highly abundant DNT, proliferating T cells, most DPT cells, and NK/ILC populations to enhance cluster resolution. (C) Dot plot illustrating the

expression of marker genes used to define the different T-cell subsets. (D) Heatmap illustrating the proportional distribution of T-cell subtypes across patients. Hierarchical clustering of patients (columns) was performed using the complete linkage method. Data represent the percentage of total T cells per patient, normalized via row Z-score scaling to compare MG and NoMG groups. (E) Stacked bar plots showing the raw cellular composition per patient, complementary to panel D but without row scaling.

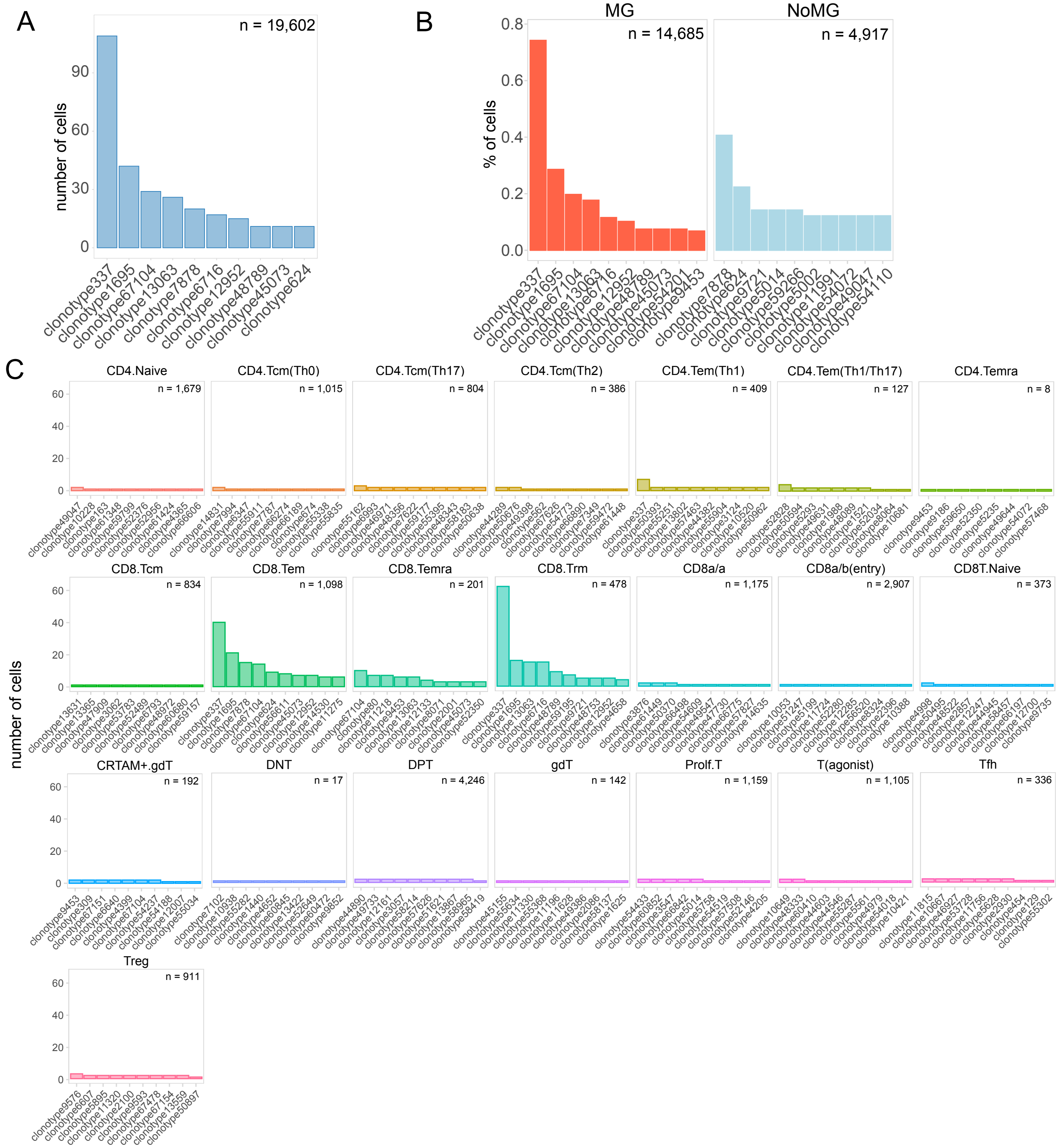

Figure S7. T-cell clonal expansion and repertoire diversity. (A) Bar plot illustrating the cell counts for the top 10 most abundant T-cell clonotypes identified across the entire dataset (n = 19,602 cells included in the clonotype analysis). (B) Comparative bar plots showing the distribution and cell counts of the top 10 clonotypes within MG and NoMG samples, respectively. (C) Bar plot illustrating the cell counts for the top 10 most abundant T-cell clonotypes for each T-cell subtype. Clonal expansion was observed in specific CD8+ T-cell subtypes, including

CD8.Tem, CD8.Temra, and CD8.Trm. These subtypes exhibit clonal expansion, characterized by multiple dominant clonotypes containing high cell frequencies per clone.

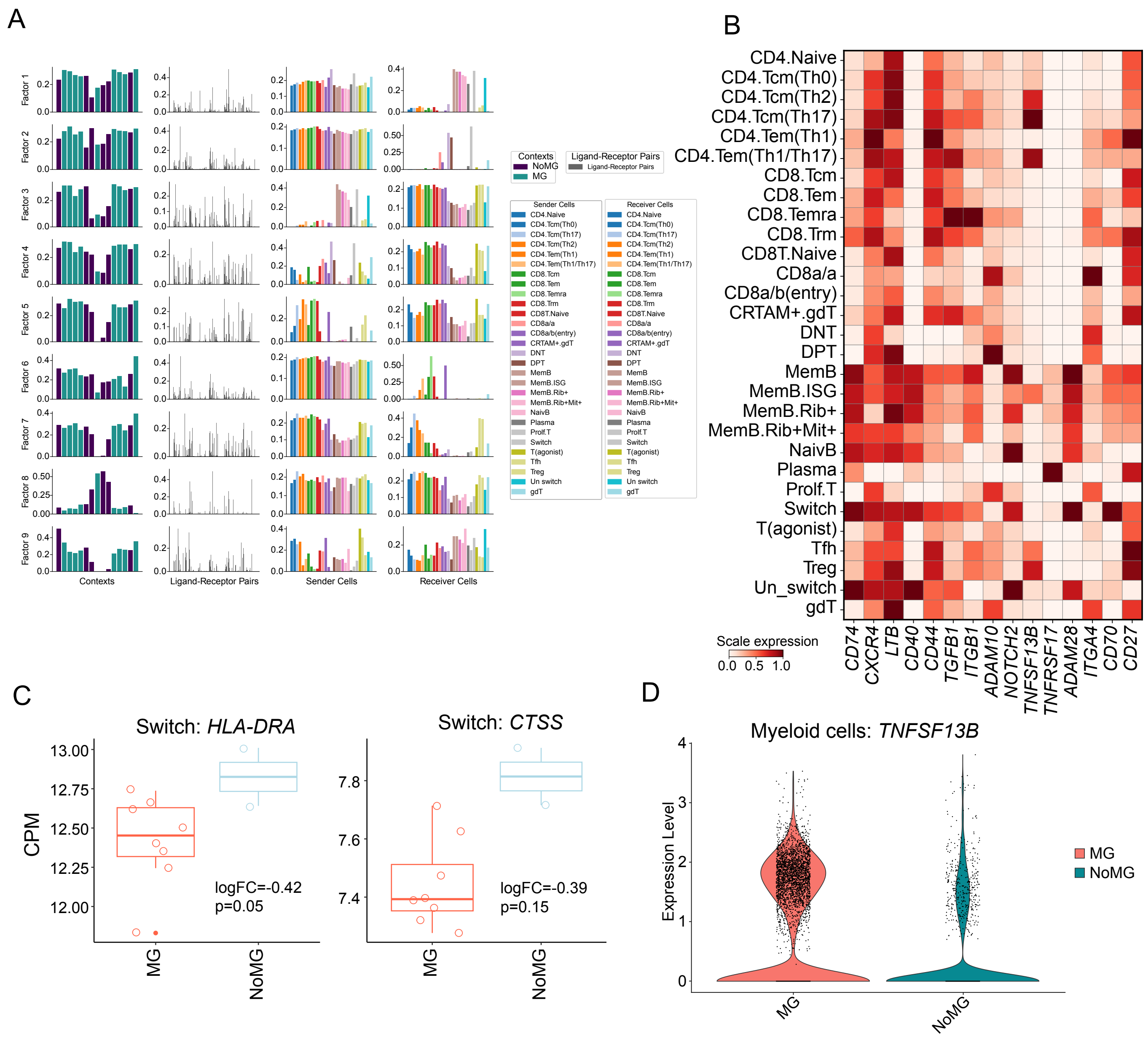

Figure S8. Altered T-B cell communication patterns in MG revealed by Tensor-Cell2cell analysis. (A) Bar plot illustrating the distribution of weight values for each factor derived from Tensor-Cell2cell analysis. Intercellular communication patterns were decomposed using the LIANA+ package (v1.5.0) via the Tensor-Cell2cell analytical framework. Weight values represent the relative contribution of each element to the identified communication modules. (B) Heatmap showing the relative expression of key ligand and receptor genes across the B- and T-cell subtypes involved in the analysis. Expression values are column-scaled (range: 0 to 1). (C) Boxplots illustrating the expression of antigen-presentation machinery (*HLA-DRA* and *CTSS*) in Switch B cells. Analysis was performed using pseudo-bulk RNA-seq profiles generated by aggregating raw single-cell counts per subject. Statistical significance was determined using the edgeR (v4.2.2) package. These data serve to

validate the differential T-B interactions; therefore, raw p-values and log2FC are reported. Each dot represents an individual subject (n = 8 MG; n = 2 NoMG), and whiskers represent 1.5x the interquartile range (IQR). (D) Violin plot demonstrating the expression levels of *TNFSF13B* (BAFF) in myeloid cells from MG and NoMG samples. The myeloid compartment comprises macrophages, neutrophils, DCs, and pDCs.

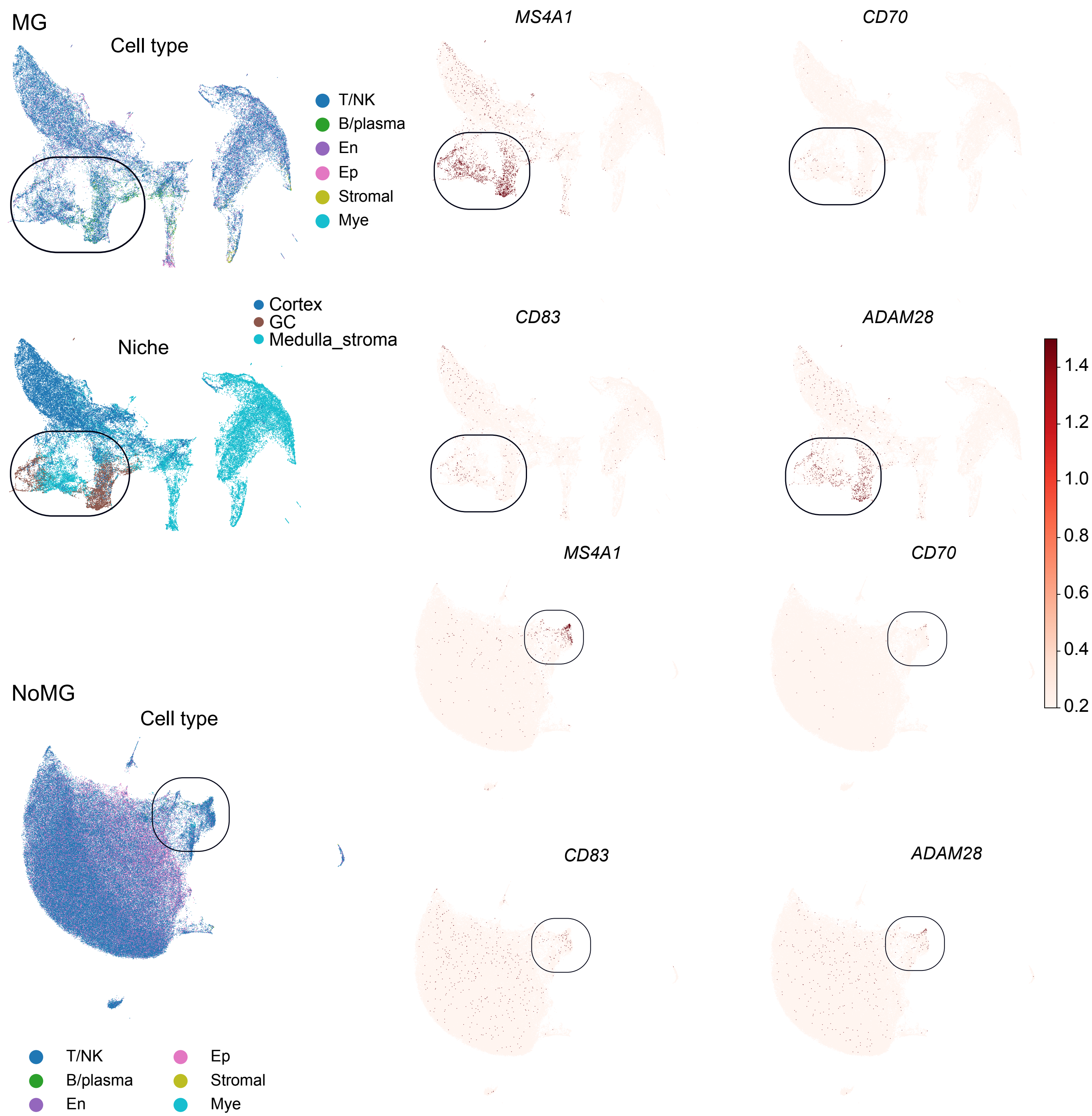

Figure S9. UMAP visualization of Switch B-cell markers and *ADAM28* expression in Visium HD data. Single-cell-like objects obtained from Bin2Cell (v0.3.3) were utilized for the analysis. UMAP projections were generated using the BANKSY package (v1.3.4), using a spatially aware clustering algorithm that incorporates the expression profiles of neighboring bins. For spatial inference, the mixing parameter (lambda) was set to 0.2 with a neighborhood size of 15. *CD70* is the key marker for Switch B cells, as identified from our scRNA-seq analysis.

A

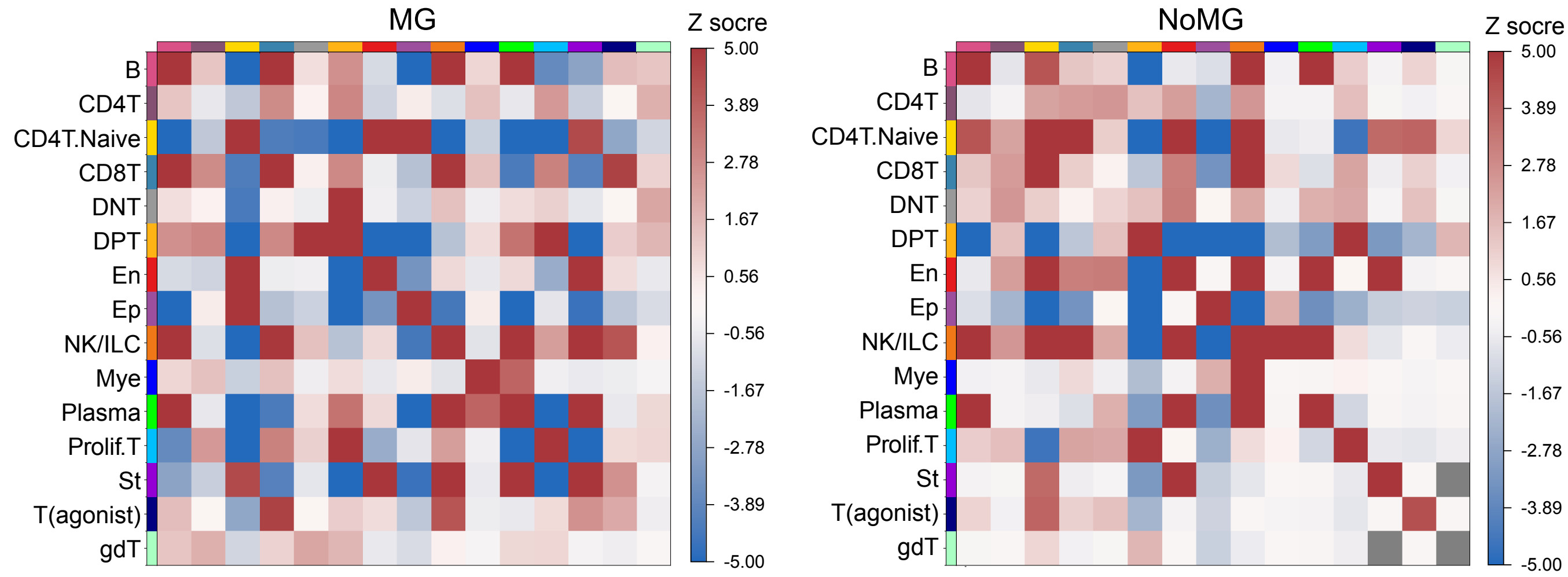

B

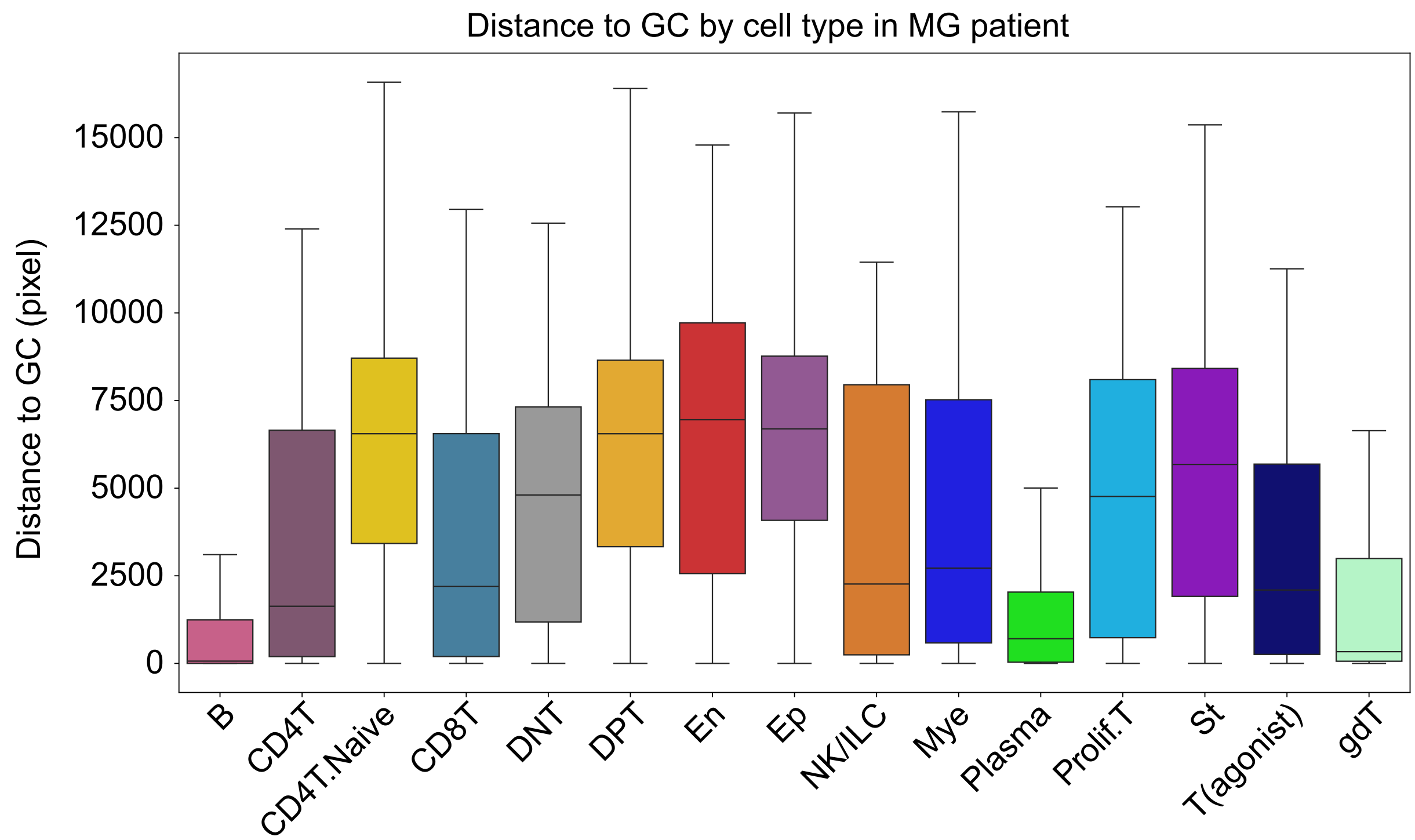

Figure S10. Spatial co-occurrence and proximity to GCs in the MG thymus. (A) Heatmap showing spatial enrichment between cell types in Visium HD data. Bin2Cell (v0.3.3) was used to aggregate 2 μm bins into single-cell-like objects for co-occurrence analysis. Neighborhood enrichment scores were calculated using the nhood_enrichment function in Squidpy (v1.6.1). Positive scores indicate spatial co-occurrence (attraction), while negative values indicate spatial exclusion. For visualization, the Z-score scale was capped from -5 to 5. (B) Boxplot illustrating the spatial distance from each cell type to the nearest GC-designated cell in the MG sample.

Distances were determined using a nearest-neighbor approach via the scikit-learn library (v1.6.1) and are expressed in spatial coordinate units (pixels). Whiskers represent 1.5x the interquartile range (IQR).

Patient #	Sample #	Specimen Type	Chemistry	Diagnosis	Antibodies	Age	Sex
PT1	S1	Thymoma w/o MG	Single Cell 3' v3	Thymoma type B2. T1b. Masaoka stage 2a. No MG	/	53	F
PT2	S2	Thymus w MG	Single Cell 3' v3	Benign thymic tissue with hyperplasia. MG. Ocular symptoms	High AchR antibodies elevated	24	F
PT3	S3	Thymus w/o MG	Single Cell 3' v3	Thymic cyst and Thymic hyperplasia. No MG	/	60	M
PT4	S4	Thymoma w/o MG	Single Cell 3' v3	No MG	/	50	F
	S5	Thymus w/o MG	Single Cell 3' v3				
PT5	S6	Thymus w MG	Single Cell 5' R2 plus V(D)J	Thymic hyperplasia, MG	AchR positive	41	F
PT6	S7	Thymus w MG	Single Cell 5' R2 plus V(D)J	Focal lymphoid hyperplasia. Ocular Myasthenia	AchR positive,	42	F
PT7	S8	Thymus w MG	Single Cell 5' R2 plus V(D)J	Thymic hyperplasia, MG	AchR negative, LRP4 positive	37	F
PT8	S9	Thymus w/o MG	Single Cell 5' R2 plus V(D)J	Thymoma B1	/	53	F
PT9	S10	Thymus w MG	Single Cell 5' R2 plus V(D)J	Thymic MG; Thymoma B2B3; metastases	AchR positive	39	F
	S11	Drop Metastases	Single Cell 5' R2 plus V(D)J				
	S12	Thymoma w MG	Single Cell 5' R2 plus V(D)J				
	S13	Metastases continuity	Single Cell 5' R2 plus V(D)J				
PT10	S14	Thymoma w/o MG	Single Cell 5' R2 plus V(D)J	Thymoma B1	/	78	M
PT11	S15	Thymus w MG	Single Cell 5' R2 plus V(D)J	Ocular MG	AchR negative, LRP4 negative, musk negative	27	F
PT12	S16	Thymus w MG	Single Cell 5' R2 plus V(D)J	Type AB thymoma	AchR positive	50	M
	S17	Thymoma w MG	Single Cell 5' R2 plus V(D)J				
PT13	S18	Thymoma w MG	Single Cell 5' R2 plus V(D)J	Thymoma AB. MG	AchR negative	53	M
PT14	S19	Thymic Carcinoma	Single Cell 5' R2 plus V(D)J	No MG	/	71	F
	S20	Thymoma (Metastatic)	Single Cell 5' R2 plus V(D)J				
PT15	S21	Thymus w MG	Single Cell 5' R2 plus V(D)J	MG, normal thymus	AchR positive	21	F
PT16	S22	Thymus w/o MG	Single Cell 5' R2 plus V(D)J	MG negative	/	71	M
	S23	Thymic Carcinoma	Single Cell 5' R2 plus V(D)J				

Table S1: Patient characteristics within the NU cohort

Yasumizu et al., 2022 (scRNA-seq dataset) (*3*)

ID	Age	Sex	anti AchR Abs titer (nmol/L)	WHO classification	Immunological pretreatment	QMG score
MG03	46	M	15	AB	No	0
MG21	55	F	1.5	B1	No	0
MG22	49	F	0.3	B2	No	0
MG23	35	F	22	AB	Methylprednisolone I.V. (250mg/day for 3days and 500mg/day for 3days in a few month before sample collection) followed by oral prednisone (5mg/day for 1.5month)	10

Xin et al., 2022 (scRNA-seq dataset)(*6*)

ID	Sex	Disease
T01	M	thymic epithelial tumor
T02	M	thymic epithelial tumor
T03	F	thymic epithelial tumor
T04	M	thymic epithelial tumor
T05	F	thymic epithelial tumor
T06	M	thymic epithelial tumor

Yasumizu et al., 2024 (Visium spatial dataset) (*2*)

Sample id	Patient id	Age	Sex	MG status	aAchr	is_Ab	Condition	WHO
TP_1_1	TP_1	55	F	seropositive_asymptomatic	1.5	T	thymoma	B1
TP_1_2	TP_1	55	F	seropositive_asymptomatic	1.5	T	thymoma	B1
TMG_1_1	TMG_1	45	F	MG	210	T	thymoma	B1
HP_1_1	HP_1	36	F	MG	1.5	T	hyperplasia	
TN_1_1	TN_1	74	F	nonMG	0	F	thymoma	B1
TMG_2_1	TMG_2	67	F	MG	5.9	T	thymoma	B2
TN_2_1	TN_2	51	F	nonMG	0	F	thymoma	B1
TN_3_1	TN_3	48	M	nonMG	0	F	thymoma	B2
HP_2_1	HP_2	24	F	MG	1.1	T	hyperplasia	
TP_2_1	TP_2	50	F	seropositive_asymptomatic	10	T	thymoma	B2

Table S2: Characteristics of the patients in public datasets
